# The use of the Auditory Processing Domains Questionnaire (APDQ) in Brazilian Portuguese with caregivers of children and adolescents with diverse neurodevelopmental conditions

**DOI:** 10.1590/2317-1782/e20240347en

**Published:** 2026-03-27

**Authors:** Joel de Braga, Claudia Maria de Lorenzo, João Carlos Xikota, Luciany Andrade Nascimento, Liliane Desgualdo Pereira, Karin Ziliotto Dias, Maria Madalena Canina Pinheiro

**Affiliations:** 1 Programa de Pós-graduação em Fonoaudiologia, Universidade Federal de Santa Catarina – UFSC - Florianópolis (SC), Brasil.; 2 Núcleo Desenvolver , Hospital Universitário Polydoro Ernani de São Thiago, Empresa Brasileira de Serviços Hospitalares – EBSERH - Florianópolis (SC), Brasil.; 3 Departamento de Pediatria, Centro de Ciências da Saúde, Universidade Federal de Santa Catarina – UFSC - Florianópolis (SC), Brasil.; 4 Departamento de Fonoaudiologia, Universidade Federal de São Paulo – UNIFESP - São Paulo (SP), Brasil.; 5 Departamento de Fonoaudiologia, Universidade Federal de Santa Catarina – UFSC - Florianópolis (SC), Brasil.

**Keywords:** Auditory Perception, Surveys and Questionnaires, Hearing, Attention Deficit Disorder with Hyperactivity, Child, Adolescent

## Abstract

**Purpose:**

To investigate the capacity of the Auditory Processing Domains Questionnaire (APDQ), in its Brazilian Portuguese version, to differentiate children and adolescents with diverse neurodevelopmental conditions based on their caregivers' responses.

**Methods:**

This was an analytical, multicenter study. Consisted of guardians of individuals aged between seven and 17 years old, divided into three groups according to neurodevelopmental condition: Control Group (CG); Human Communication Disorders Group (HCDG); and Attention Deficit Hyperactivity Disorder Group (ADHDG). All caregivers completed the APDQ, a scale consisting of 50 questions divided into three domains. The questionnaire generates a percentage score for each domain, a total score, and an indication of risk for developmental impairment, referred to as the outcome. The data was analyzed descriptively and analytically, considering a significance level of 5% (p<0.05).

**Results:**

There was a significant difference between the groups in the total score and in the APDQ domains, indicating that the instrument can differentiate between children with and without neurodevelopmental disorders. In the attention domain, there was a significant difference between ADHDG and HCDG, distinguishing the performance between these groups. In terms of outcomes, the APDQ was able to differentiate between the groups, classifying the majority of CG individuals as “normal listening”, and those from HCDG and ADHDG as being at risk of “language, learning and listening difficulties”.

**Conclusion:**

The use of the APDQ was effective in distinguishing the different neurodevelopmental conditions. The attention domain stood out, which enabled to differentiate between HCDG and ADHDG.

## INTRODUCTION

Central Auditory Processing Disorder (CAPD) refers to an alteration in one or more auditory processing abilities, which may result in difficulties in speech comprehension in noisy environments, in the interpretation of simple and complex instructions, and it affects nonverbal aspects, negatively affecting learning, including reading and writing skills^(1,2)^.

Currently, the prevalence of CAPD in children is a relevant issue, with percentages varying across diverse populations^(3,4)^. In the United States and the United Kingdom, the prevalence has been estimated between 2-7%^(4,5)^; in New Zealand, the overall prevalence in childhood has been estimated at 6.2%^(6)^, while studies suggest that 3-7% of school-aged children face learning difficulties^(3)^. Therefore, a significant number of children are referred for assessment^(6)^. This increase in referrals for assessment can be attributed to greater awareness of CAPD, the use of subjective questionnaires for screening, and the impact the disorder can have on children's academic and social lives.

Therefore, early identification of CAPD in children is essential to ensure timely referral for assessment and intervention, accelerating the initiation of therapeutic treatment and reducing the risk of academic and social difficulties^(2,6,7)^. Furthermore, the earlier CAPD is detected, the more quickly modifications can be implemented in the school and home environments, promoting a context that favors the child's learning, understanding, and communication. Early interventions also take advantage of brain plasticity, as children's brains are still maturing, facilitating cognitive development and preventing the worsening of difficulties^(2,8,9)^.

CAPD is associated with a range of signs and symptoms, including difficulties with attention and distractibility, reading, spelling and/or learning problems^(1)^; among the comorbidities, Attention Deficit/Hyperactivity Disorder (ADHD) has been particularly highlighted. Studies have shown the importance of assessing central auditory processing (CAP) in children with ADHD to contribute to complementary diagnosis and rehabilitation^(10,11)^. The relationship between ADHD and CAPD is significant, as both conditions frequently co-occur and manifest in early childhood with overlapping complaints related to attention, memory, difficulty sustaining concentration, and following auditory instructions. Such symptom overlap poses a challenge for the clinical diagnosis of CAPD in children with ADHD as well as for the identification of ADHD in children with CAPD. Therefore, research is being conducted to investigate screening methods for CAPD early identification in individuals at-risk, to help differentiate between these diagnoses that may present similar symptoms^(10,12)^.

Internationally, guidelines indicate various instruments as a way to screen for potential risks and/or behavioral manifestations related to CAPD, such as scales, questionnaires, and/or auditory test batteries. These tools are potentially useful in identifying auditory disabilities in diverse study populations. The documents state that questionnaires provide information about auditory behavior and its impact on communication, academic performance, or work, and are strongly suggested in clinical practice for early identification and intervention in children at risk for CAPD^(2,6,9,13)^.

In the Brazilian literature, instruments for the pediatric population include informally developed questionnaires lacking validation; translated and validated questionnaires; and questionnaires that have only been translated, without validation data derived from representative samples across different populations. These instruments are: *Scale of Auditory Behaviors (SAB)* ^(14)^; *Fisher's auditory problems checklist (QFISHER)* ^(15)^; *Children's Auditory Processing Performance Scale* (CHAPS)^(16)^; and the *Auditory Processing Domains Questionnaire (APDQ)* ^(17)^.

Among these questionnaires, the APDQ is relatively recent in the literature and, to date, it has a limited number of publications^(18-21)^. However, the APDQ has been widely recommended due to its high methodological rigor and adequate level of sensitivity and specificity, which supports its use in research applications^(22)^. Developed by O'Hara and Mealings to assist in the diagnosis of CAPD, the APDQ allows the identification of individuals at risk for CAPD or at risk for other comorbidities. The APDQ is an appropriate instrument for parents/guardians to report their perception of the language, attention, and everyday auditory abilities of individuals between 7 and 17 years^(17)^.

In Brazil, Dias et al.^(23)^ conducted a study involving the translation, cross-cultural adaptation, and evaluation of the reliability and internal validity of the 52-question instrument in 7-to-17 -year normal-hearing individuals, without risk factors for CAPD. The results demonstrated that the instrument presents favorable translation quality, internal validity, and reliability for its use^(23)^. However, in 2021, Brian O'Hara, one of the questionnaire's author, revised the questions and proposed reducing the APDQ by two questions, resulting in a total of 50 questions^(24)^. The new version with 50 questions was also recently analyzed and published in a Brazilian study, which described the translation and adaptation process of the APDQ into Brazilian Portuguese. The article presented the changes made comparing the two versions of the questionnaire - such as the reformulation of the wording and the exclusion of some questions -, detailed the entire methodology for application and handling of the spreadsheet, and applied the instrument to a small pilot group^(25)^. In addition, a Brazilian study used the new version with individuals with Autism Spectrum Disorder (ASD) Level 1 and neurotypical participants^(26)^.

Considering that the APDQ questionnaire differentiates aspects of attention and language, the use of the new version proposed by the author, which reduced the number of questions from 52 to 50, will make a significant clinical and scientific contribution. This will assist in the screening, diagnosis, and rehabilitation of children with CAPD, in addition to facilitating the identification and differentiation of the CAPD signs and symptoms in relation to other clinical comorbidities, such as ADHD. Thus, the objective of this study was to investigate the ability of the APDQ, in its Brazilian Portuguese version, to differentiate children and adolescents with different neurodevelopmental conditions based on their caregivers’ responses.

## METHOD

This is an observational, analytical, cross-sectional, and multicenter study involving three institutions in southern and southeastern Brazil. This study was approved by the Research Ethics Committee with Human Beings (RECHB), under number 5.268.520. All guardians of the children and/or adolescents were initially informed about the objectives of the study and, after the explanation, signed the Informed Consent Form specific to the legal guardians of minors, authorizing their participation in the research. For the minors, they were asked to sign the Informed Assent Form.

This study continues the research on the APDQ in its Brazilian version. With the reduction of the instrument to 50 questions and the improvement of the wording of the questions, the questionnaire became more accessible and understandable for the target audience. The article detailing the entire process of translation, adaptation, application, and analysis of the APDQ from Brazilian Portuguese with its 50 questions is currently published in a scientific journal^(25)^.

### Sample definition

This is a convenience sample composed of three groups. The following describes how each group was formed and recruited.

### Inclusion and exclusion criteria

The inclusion criteria listed for the study were: caregivers of individuals aged between seven and 17 years, of both biological sexes, with Brazilian Portuguese as their mother tongue, absence of middle ear pathologies, hearing thresholds within normal ranges bilaterally^(27)^.

The exclusion criteria included the presence of reports obtained during the anamnesis, of neurological alterations in the research participants, and/or evaluators’ observation of evident cognitive impairments.

For the **Control Group (CG)**, participants were caregivers of individuals with no history of middle ear disorders in childhood, good academic performance reported in their medical history, and absence of dyslexia, ADHD, or any other neurodevelopmental disorder. Participants completed a minimal battery of behavioral tests, the results of which indicated auditory abilities within the expected range for the^(28,29)^ age group. In addition, CG individuals had to present a total score on the SAB higher than or equal to 46 points^(14)^ and, had not marked six or more items as 'quite a lot' and/or 'too much' in items 1 to 9 and/or 10 to 18^(30)^ on the SNAP-IV (Swanson, Nolan, and Pelham Rating Scale).

The **ADHD Group (ADHDG)** was formed by caregivers of individuals with a multidisciplinary diagnosis of ADHD, which could be of the inattentive, hyperactive, or combined type, confirmed through multidisciplinary assessment. Individuals who had a score of 6 points or higher on questions 1 to 9 or 10 to 18 on the SNAP-IV questionnaire were included. In addition, they could also present with some human communication disorder, reported by the caregivers in the anamnesis, and a score on the SAB lower than 46 points and/or altered auditory processing abilities in the CAP assessment.

In the **Human Communication Disorder Group (HCDG)**, caregivers of individuals with reading, writing, and/or speaking difficulties, and/or alterations in at least one of the tests in the minimum CAPD battery were included^(28,29)^. Furthermore, all individuals had to present some human communication disorder (oral and/or written language impairments), except for ADHD. To exclude signs and symptoms suggestive of ADHD, only caregivers of individuals who did not have a score of 6 points or higher on questions 1 to 9 or on questions 10 to 18 on the SNAP-IV questionnaire were included^(30)^. In addition, they had to present a score of less than 46 points on the SAB^(14)^.

### Assessments

All individuals in the sample underwent a minimum battery of CAPD assessments, and CAPD was considered present when there was an alteration in one or more of the assessed auditory abilities^(1,2,31)^. The minimum battery was developed based on national and international recommendations and guidelines, including the following tests^(1,2,31)^: **temporal processing**: Random Gap Detection Test (RGDT)^(32)^ or Gaps in Noise (GIN)^(33)^ in the assessment of temporal resolution, with GIN performed in only nine patients, two from the ADHDG and seven from the HCDG, as they were adolescents; and the frequency pattern test for assessing temporal ordering, with the Auditec version^(34)^ applied to individuals up to 9 years and 11 months old, and the Musiek version^(35)^ for 10 years or older; **Sound recognition in a dichotic task:** dichotic digit test (binaural integration and separation)^(28)^; **Recognition of verbal sounds in a monotic task**: Pediatric Speech Intelligibility Test (PSI)^(28)^ or the Synthetic Sentence Identification Test in Monotic Hearing (SSI)^(28)^, at signal-to-noise ratios of 0, -10 and -15 dB. The SSI was applied to individuals who demonstrated fluent reading proficiency. In addition to the **simplified assessment** ^(28)^ to complement the assessment (sound localization in five directions, verbal and nonverbal sequential memory test).

In addition, all individuals’ guardians completed the SNAP-IV questionnaire^(30)^, an instrument that provides information about inattention and/or hyperactivity, widely used by professionals, and the SAB questionnaire^(14)^, an instrument composed of 12 closed-ended questions that investigate behavioral hearing difficulties perceived in the individuals' daily lives.

### Division of groups

Based on the inclusion and exclusion criteria and the applied assessments, participants were distributed into three groups:

**CG:** comprised of caregivers of individuals with a history of typical neurodevelopment, with CAP assessments within normal range for their age group and no complaints in the anamnesis and/or in the SAB^(14)^ and SNAP-IV^(30)^ questionnaires. Group members were recruited by the researchers through an electronic form distributed on social networks and messaging groups.
**ADHDG:** comprised of caregivers of individuals with a multidisciplinary diagnosis of ADHD and with complaints reported in anamnesis and in the SAB^(14)^ and SNAP-IV^(30)^ questionnaires. Participants were recruited through a partnership established for research purposes with the pediatric outpatient clinic of a university hospital in the south of the country. Individuals from the outpatient clinic underwent a multidisciplinary assessment and, after diagnosis, those who met the inclusion criteria were invited by the head researcher to participate in the study.
**HCDG:** comprised of caregivers of individuals with speech, reading, and/or writing difficulties reported in anamnesis and/or the SAB^(14)^ questionnaire, and altered auditory abilities. Group members were recruited from two institutions located in the southeast of the country, in addition to those receiving care in an outreach project and during internships in a speech-language pathology course in the south of the country.

### APDQ application

The APDQ questionnaire has three domains analyzed in a complex manner, allowing the identification of possible deficits in auditory processing, attention and/or language in the individual being assessed, directing the referral to a specialized professional (Annex A).

The questionnaire was administered by the researchers to the individuals' guardians, and the questionnaire took approximately 30 minutes to complete.

The APDQ applied in this study involves 50 questions grouped into three domains: auditory processing with 29 items; attention with 10 items; and language with 11 items. Each question has five response options with the following scores: Four points for behavior that occurs almost always; Three points for frequent behavior; One point if it occurs sometimes; Zero points if the behavior rarely occurs; “Not applicable” receives no score and the question is excluded from the final calculation.

The percentage values can be obtained by calculating the following formula:


$$Score\;=\;\frac{points\;obtained\;in\;items\;of\;a\;certain\;domain}{4\;\times \;number\;of\;maximum\;points\;of\;that\;domain}\times 100$$ (1)

The maximum points that can be obtained in each domain is 116 points for auditory processing (29 questions), 40 points for attention (10 questions), and 44 points for language (11 questions), totaling 200 points, which suggests the absence of a possible risk for neurodevelopmental disorders. However, the total questionnaire score can reach 204 points, since question 3 is present in two domains, while question 2 is not associated with any of the domains but can be counted in the total score.

The APDQ includes a Microsoft Excel spreadsheet denominated “*Database*”, available on the questionnaire author's website, along with a handbook^24.^ This spreadsheet was programmed by the questionnaire author to perform the rather complex calculations and generate the final report, indicating the percentage in each domain and the primary risk of each individual, which can be classified into one of eight risk categories: High risk of CAPD; Risk of CAPD; High risk of ADHD; Risk of ADHD; Combined risk of CAPD and ADHD; Difficulties in listening, learning and language; Deficits in language and normal listening.

### Interpretation of the APDQ

In the present study, the cut-off points (Chart 1 and Table 1) established in the original study by Brian O'Hara and Mealings^(17)^ were followed. Criteria 1) and 2) presented below are the same as in the original questionnaire study, since, to date, no Brazilian study presents specific values for the population of Brazilian children and adolescents in the current version of the questionnaire.

**Chart 1:** Risk percentages and percentiles for alterations in Auditory Processing Domains Questionnaire

**Percentile**	**Age range**	**Auditory processing**	**Attention**	**Language**
15th to 20th	7 to 10 years	≤ 70%	≤ 60%	≤ 80%
Lower risk	11 to 17 years old	≤ 78%	≤ 67%	≤ 84%
5th to 10th	7 to 10 years	≤ 56%	≤ 42%	≤ 72%
Higher risk	11 to 17 years old	≤ 62%	≤ 53%	≤ 78%

Source: O'Hara and Mealings^(17)^

**Table 1:** Summary of percentiles needed to differentiate the risks proposed by the Auditory Processing Domains Questionnaire

**Primary risk**	**Percentile**			**Difference between ATT and AP**
	**AP**	**ATT**	**LGG**	
High risk of CAPD	≤ 5°	-	≥ 3°	≥ 0
Risk of CAPD	≤ 15	-	≥ 3°	≥ 0
High risk of ADHD	-	≤ 10* / ≤ 5**	≥ 3°	≤ -9
Risk of ADHD	-	≤ 20	≥ 3°	≤ -9
Combined risk of CAPD and ADHD	≤ 15	≤ 20	≥ 3°	-1 to -8
Difficulties in listening, learning, and language.	-	-	≤ 3°	-
Language deficits	-	-	3rd to 15th	-
Normal listening	> 15°	> 20	>15	-

* Suggested percentile for younger individuals (7 to 10 years);

** Suggested percentile for older individuals (11 to 17 years)

**Caption:** AP = Auditory Processing; ATT = Attention; LGG = Language; CAPD = Central Auditory Processing Disorder; ADHD = Attention Deficit Hyperactivity Disorder. Source: O'Hara and Mealings^(17)^

To differentiate between the potential risks an individual may present, the questionnaire is based on two criteria:

1. **Cut-off point:** In each domain, a percentile indicates whether the individual is at risk and whether that risk is higher or lower. The values are organized in Chart 1.
2. **Difference between the attention domain and the auditory processing domain:** this difference aids in identifying the risk individuals present. The difference between the scores of the attention and auditory processing domains suggests the following risks:

- Scores equal to or above 0: If the difference between the scores for the attention and auditory processing domains is equal to or higher than 0, it suggests that the person may be experiencing difficulties related to auditory processing.
- Scores between -1 and -8: If the difference between the scores in the attention and auditory processing domains is between -1 and -8, this may indicate combined risk factors for CAPD and ADHD.
- Scores below -9: If the difference between the scores in the attention and auditory processing domains is below -9, the individual may be at risk of ADHD.

Primary language risk constitutes a specific case within the APDQ framework. To be classified as having a primary risk of language deficit, an individual must obtain a score of 45% or lower (below the 3rd percentile). This indicates these individuals scored very low on language-related items, and therefore a more pronounced impairment in this domain. Consequently, the identification of primary risks of CAPD and/or ADHD is precluded, as these classifications require sufficient performance in the language domain. This distinction is an important consideration when prioritizing referrals based on APDQ results.

These guidelines in the APDQ handbook are based on data collected by the author to define the cut-off criteria. The construction of these criteria involved analyzing the responses of 190 individuals in the control group, 20 students diagnosed with auditory processing disorder, 45 students with attention deficit disorder, and 18 students with unspecified learning disabilities, all attending special education classes^(17,24)^. The results obtained in the three assessed domains - auditory processing, language, and attention - indicate the possible primary risks identified by the APDQ.

Cut-off points were defined through statistical analyses, such as the Receiver Operating Characteristic Curve (ROC curve) and linear regression, which established percentages and percentiles associated with the risk of alteration. In the original study, external validity analyses, performed with the linear regression model, revealed statistically significant differences between the group with typical development and the clinical groups on all scales (p < 0.001), in addition to significant differences between the clinical groups themselves. The ROC curve identified cut-off points with sensitivity and specificity levels ranging from 80% to 90%. Cut-off points established between the 15th and 20th percentiles prioritize sensitivity and are highlighted in yellow in the reports. Cut-off points between the 5th and 10th percentiles prioritize specificity and are highlighted in red^(17)^.

Table 1 summarizes the percentiles criteria required for the questionnaire to differentiate individuals across the eight possible primary APDQ risk categories.

### Statistical analysis

For the categorical variables in the sample, the data were represented using absolute and relative frequencies. The numerical variables were described using measures of dispersion.

Regarding age range, individuals were stratified into two groups, according to the questionnaire author's proposal: children aged 7 to 10 were classified as "younger children," and those aged 11 to 17 as "older children."

Quantitative variables were assessed for normality using the Shapiro-Wilk test, skewness, kurtosis, and histograms. When the distribution was normal, the independent samples t-test was applied. When normality assumptions were not met, the non-parametric Mann-Whitney and Kruskal-Wallis tests were used. When the Kruskal -Wallis test indicated a statistically significant difference (p < 0.05), a Dunn's post-hoc test was performed for multiple comparisons. Only comparisons that remained statistically significant in the post-hoc analysis were considered significant^(36)^.

In the analysis of the eight outcomes, two of them were grouped to avoid excessive data dispersion, where each item would present very small values. Thus, "High risk of CAPD" and "risk of CAPD" were combined in outcome 1; "High risk of ADHD" and "risk of ADHD" formed outcome 2; "Combined risk of CAPD and ADHD" formed outcome 3; "Difficulty in language, learning and listening" formed outcome 4; "Language deficits" formed outcome 5, and "Normal listening" outcome 6. To assess the difference of the six outcomes between the groups, Fisher's Exact test was used. When an association was found, the difference between the categories was investigated by the adjusted standardized residuals, whose value above 2.0 standard deviations indicated the cells/boxes with a difference between the percentages and signaled in the table with the letters "a, b, c, d". The data were stored in Microsoft Excel spreadsheets and subsequently exported to the software Stata, version 14.0. For statistical analyses, a significance level of 5% (p < 0.05) was considered.

## RESULTS

In the composition of the individuals according to the groups, it was observed that 19 individuals were excluded for not meeting the inclusion criteria. In the end, 118 individuals were selected, the majority (54.2%) between 7 and 10 years (younger children), 58.5% were male, and 17.79% attending the 5th grade of elementary school.

Table 2 presents the data characterizing the sample in relation to sociodemographic data and hearing impairments according to the study groups. It should be noted that information regarding the educational level of four caregivers could not be obtained, as the family member responding to the questionnaire was unaware of this information. Regarding the CAP assessment, all individuals in the ADHD group presented at least one altered auditory processing ability, and this group exhibited a greater number of auditory impairments compared to the HCDG.

**Table 2:** Characterization of the sample according to socio-demographic data and hearing impairments

**Variables**	**CG (n = 30)**	**HCDG (n = 38)**	**ADHDG (n = 50)**
**Age (years)**			
Mean	9.5	11	10
Min- Max (SD)	7 - 16 (2.7)	7 - 17 (2.7)	7 - 17 (2.1)
**Sex**			
M (n - %)	13 (44%)	22 (57%)	34 (68%)
F (n - %)	17 (56%)	16 (43%)	16 (32%)
No hearing impairments			
1 to 2 disabilities	0	8 (21.06%)	6 (12%)
3 to 4 disabilities	0	14 (36.84%)	22 (44%)
5 or more disabilities	0	16 (42,10%)	22 (44%)
**Father's education level**			
Primary education (n - %)	1 (3.3%)	12 (32.43%)	15 (31.91%)
High school (n - %)	10 (33.3%)	14 (37.85%)	30 (63.85%)
Higher education (n - %)	14 (46.7%)	9 (24.32%)	1 (2.12%)
Graduate studies (n - %)	5 (16.7%)	2 (5.40%)	1 (2.12%)
**Mother's education level**			
Primary education (n - %)	0	4 (10.54%)	12 (24%)
High school (n - %)	7 (24.1%)	21 (55.26%)	25 (50%)
Higher education (n - %)	17 (58.7%)	10 (26.31%)	10 (20%)
Graduate studies (n - %)	5 (17.2%)	3 (7.89%)	3 (6%)

**Caption:** CG = Control Group; HCDG = Human Communication Disorders Group; ADHDG = Attention Deficit Hyperactivity Disorder Group; N = number of participants; M = Male; F = Female; % = percentage; Min = Minimum; Max = maximum; SD = standard deviation

Table 3 presents the performance of the three groups on the minimum CAP battery, exclusively to characterize the sample and complement the data. It was observed that, in most tests, the ADHDG had the worst performance, followed by the HCDG and CG.

**Table 3:** Descriptive analysis of the minimum central auditory processing battery according to the study and control groups

**Behavioral tests**			**CG**		**ADHDG**		**HCDG**	
			**Mean (SD)**	**Median**	**Mean (SD)**	**Median**	**Mean (SD)**	**Median**
**Sound localization (n)**			4.73 (0.44)	5	4.48 (0.73)	**5**	4.71 (0.56)	**5**
**Verbal sequential memory test (n)**			2.96 (0.18)	3	1.90 (1.02)	2	2.23 (0.75)	2
**Nonverbal sequential memory test (n)**			2.73 (0.44)	3	2.09 (0.85)	2	2.28 (0.83)	2.5
**RGDT (ms)**			6.04 (2.97)	5.75	64.50 (95.63)	22.50	65.13 (101.14)	14
**GIN (ms)**	**RE**		**-**	**-**	7.5 (6.36)	7.5	6.85 (3.18)	5
	**LE**		**-**	**-**	10 (7.07)	10	6.85 (2.73)	6
**Dichotic digits test - integration (%)**	**RE**		97.17 (0 04)	100	89.11 (11.02)	93.13	91.25 (8.66)	94.38
	**LE**		96.92 (0.04)	97.50	86.33 (11.93)	87.50	90 (7.70)	93
**Dichotic digit test - separation (%)**	**RE**		94.08 (0.07)	95	82.98 (12.38)	87.50	83.41 (15.43)	85
	**LE**		92.83 (0.07)	95	75.59 (16.09)	16.09	77.16 (19.43)	82.50
**Pitch Pattern Sequence - Auditec (%)**	**Naming**		91.55 (0.07)	93.33	62 (32.35)	73	74 (20.29)	77
	**Humming**		**-**	**-**	54 (36.06)	65	79 (13.26)	80
**Pitch Pattern Sequence - Musiek (%)**	**Naming**		91.68 (0.08)	93.33	44 (33.53)	46	53.84 (30.35)	60
	**Humming**		-	-	53.84 (30.35)	60	51.10 (27.65)	77
**Pediatric Speech Intelligibility Test - PSI (%)**	**RE**	SNR (0)	93.48 (0.77)	100	89 (12.46)	90	90 (15.21)	100
		SNR (-10)	89.09 (0.10)	90	83 (15.01)	80	84.24 (15.01)	90
		SNR (-15)	75.83 (0.10)	75	65 (17.96)	60	66.54 (23.82)	70
**Pediatric Speech Intelligibility Test - PSI (%)**	**LE**	SNR (0)	93.21 (0.07)	100	90 (9.62)	90	87.58 (17.14)	90
		SNR (-10)	92.73 (0.08)	90	87 (14.89)	90	86.67 (16.14)	90
		SNR (-15)	73.33 (0.12)	75	76 (19.83)	70	81.54 (25.41)	95
**Synthetic Sentence Identification Test - SSI (%)**	**RE**	SNR (0)	91.67 (0.09)	95	80 (12.25)	80	72.50 (22.17)	70
		SNR (-10)	90 (0.10)	90	82 (17.22)	85	66 (28.81)	60
		SNR (-15)	78.33 (0.07)	80	70 (18.71)	70	53.33 (32.15)	40
	**LE**	SNR (0)	91.67 (0.11)	95	76 (18.17)	70	70 (25.82)	70
		SNR (-10)	91.67 (0.11)	95	82 (19.41)	85	58 (27.75)	60
		SNR (-15)	86.67 (0.10)	90	86 (13.42)	86	73.33 (20.82)	80

**Caption:** n = absolute number; % = percentage; ms = milliseconds; RE = Right ear; LE = Left ear; SD = Standard deviation; CG = Control group; ADHDG = Attention Deficit Hyperactivity Disorder group; HCDG = Human Communication Disorder group; RGDT = random gap detection test; Gaps in noise (GIN); SNR = signal-to-noise ratio

Table 4 presents the total APDQ scores according to age groups of the individuals whose respondents comprised the study groups. The individuals were stratified into "younger children" and "older children"; however, no statistically significant differences were observed between age groups and the questionnaire score, either for the total sample or for the study groups.

**Table 4:** Description of total APDQ score among age groups according to study groups and total sample

**Variable**	**Total APDQ score**		
	**Younger children (7 to 10)**	**Older children (11 to 17)**	**p-value**
**CG *(n=30)***	n = 20	n = 10	0.643^a^
Mean (SD)	174.4 (26.8)	166.1 (36.1)	
Median (IQR)	180.5 (162 – 191.5)	181 (164 – 182)	
**HCDG *(n=38)***	n = 17	n = 21	0.511^b^
Mean (SD)	106.1 (46.8)	114.9 (34.1)	
Median (IQR)	124 (72 – 132)	117 (80 – 144)	
**ADHDG *(n=50)***	n= 26	n = 24	0.144^b^
Mean (SD)	83.6 (24.5)	95.4 (31.6)	
Median (IQR)	77 (64 – 97)	91 (70 – 123)	
**Total sample size *(n=118)***	n= 63	n = 55	0.963^a^
Mean (SD)	118.0 (50.7)	116.0 (41.6)	
Median (IQR)	114 (74.5 – 167.5)	116.5 (80 – 147)	

a Mann-Whitney test;

b Student's t-test

**Caption:** SD = Standard deviation; IQR = Interquartile range; APDQ = Auditory Processing Domains Questionnaire; CG = Control Group; HCDG = Human Communication Disorder Group; ADHDG = Attention Deficit Hyperactivity Disorder Group; N = number of participants

**Figure 1:**
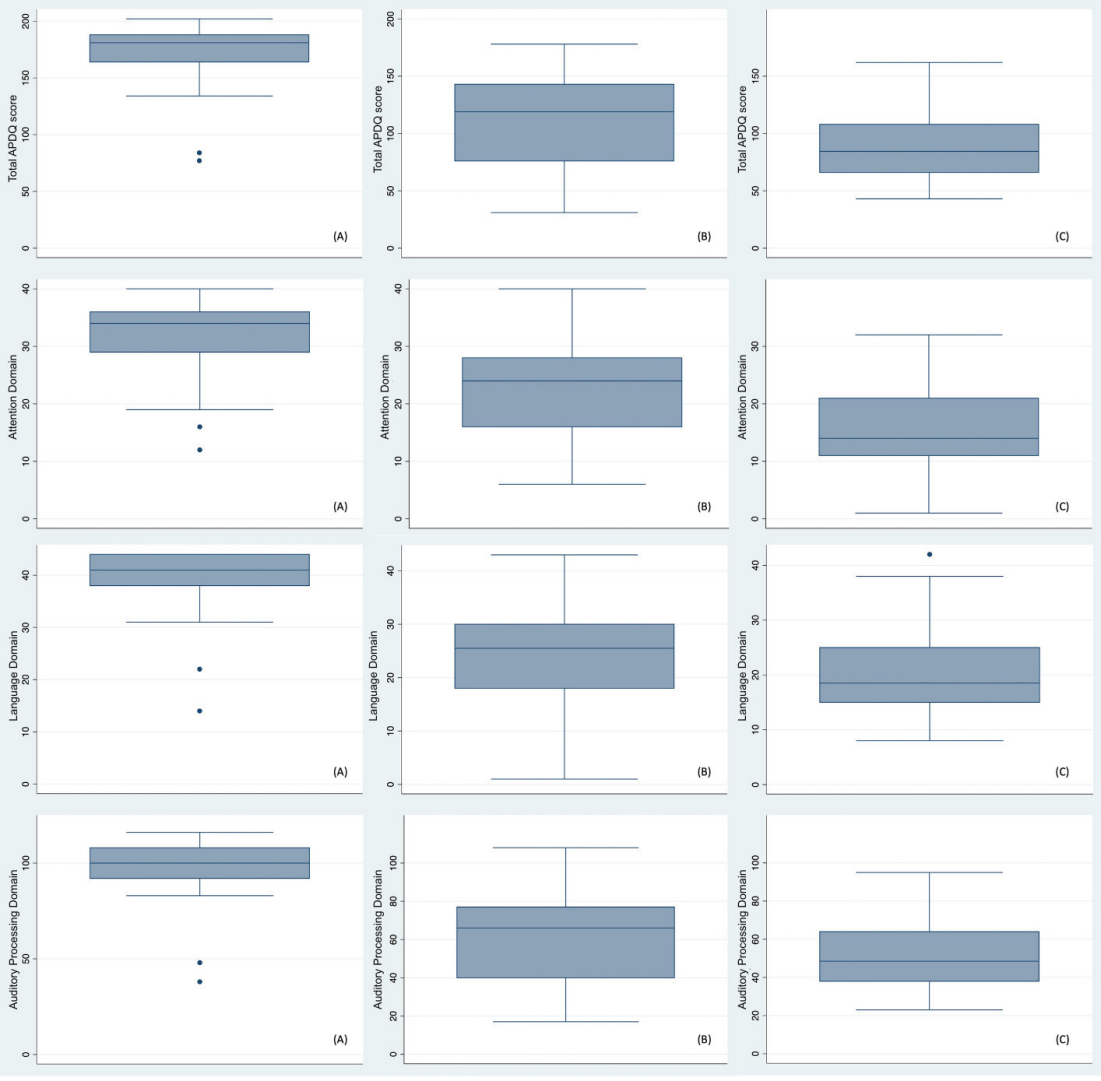
Description of APDQ total score and domains

Table 5 and Figure 1 present the total APDQ score according to the domains in each group. It is seen that the control group presented higher medians, both in the total APDQ score and in the various domains of the instrument, when compared to the HCDG and ADHDG, this asymmetry statistically significant (p = 0.001) in the Kruskal-Wallis test.

**Table 5:** Analysis of APDQ total score and domains by group

**Domains**	**CG (n=30)**	**HCDG (n=38)**	**ADHDG (n=50)**	**p-value**	**p-value** *
**Total score**				**< 0.001**	
Mean (SD)	171 (29.9)	111 (40.0)	89 (28.3)		**a,b - < 0.001**
Median (IQR)	181 (164 - 188) a,b	119 (76 - 143)a, b, c	84.5 (66 - 108) a, b, c		c > 0.079
**Attention**				**< 0.001**	
Mean (SD)	31.8 (7.28)	22.3 (8.3)	15.4 (7.6)		**a,b - < 0.001**
Median (IQR)	34 (29 - 36) a, b	24 (16 - 28) a, b, c	14 (11 - 21) a, b, c		**c > 0.005**
Percentile					
7 to 10 years (younger)	84%	47%	24%		
11 to 17 years (older)	78%	53%	28%		
**Language**				**< 0.001**	
Mean (SD)	39.2 (6.9)	24.2 (10.2)	20.6 (8.2)		**a,b - < 0.001**
Median (IQR)	41 (38 - 44) a, b	25.5 (18 - 30) a, b, c	18.5 (15 - 25) a, b, c		c > 0.306
Percentile					
7 to 10 years (younger)	99%	56%	42%		
11 to 17 years (older)	92%	55%	46%		
**Auditory processing**				**< 0.001**	
Mean (SD)	96.8 (17.3)	62.2 (23.5)	51.4 (17.5)		**a,b - < 0.001**
Median (IQR)	100 (92 - 108) a, b	66 (40 - 77) a, b, c	48.5 (38 - 64) a, b, c		c > 0.154
Percentile					
7 to 10 years (younger)	87%	55%	40%		
11 to 17 years (older)	86%	54%	47%		

Kruskal -Wallis test

* Dun 's post-hoc test

**Caption:** APDQ = Auditory Processing Domains Questionnaire*;* n = number of participants; CG = Control Group; HCDG = Human Communication Disorders Group; ADHDG = Attention Deficit Hyperactivity Disorder Group; SD = standard deviation; IQR = Interquartile Range

**Caption:** Med = median; A = control group; B = human communication disorder group; C = ADHD group. *Kruskal-Wallis test

During the post-hoc test for comparisons between groups according to total score and domains, it was found that the differences were statistically significant in the comparisons between the ADHDG and the CG (p = 0.001), as well as between the HCDG and the CG (p = 0.001). No significant differences were identified between the ADHDG and the CG in the total questionnaire score, nor in the language and auditory processing domains (p > 0.05). The only exception was observed in the attention domain, in which there was a difference between the two groups (p = 0.005), indicating that, in this domain, the questionnaire not only distinguished the control group from the study groups, but also differentiated the two study groups from each other.

Regarding the APDQ percentage values presented in Table 5, it is observed that the CG showed the highest values in all three domains, both among younger and older participants. The language domain showed the highest percentages, followed by auditory processing, and lastly, attention, with the lowest values. In the HCDG, the auditory processing and language domains showed similar percentages, slightly higher than those of the attention domain. In the ADHDG, the auditory processing and language domains showed similar values, but with a more pronounced difference in relation to the attention domain, which showed the lowest values. Regarding percentages, the CG did not present a risk of alteration, unlike the other groups, which, when compared to the cutoff values (Chart 1), presented percentile scores indicating a risk of alteration.

Chart 2 describes the APDQ outcomes according to the groups. It can be observed that, in the control group, 70% of cases exhibited an outcome 6. For the HCDG, the largest proportion of the sample was classified with outcome 4 (31.6%), followed by outcome 1 (26.3%). In the ADHDG, the highest occurrences were outcome 4 (54.0%) and outcome 2 (22%). There was a difference in the proportions of outcome 1 (3.3% *vs* 26.3%) and outcome 6 (70.0% *vs* 2.6%) between the CG and the HCDG. Differences were also observed in the proportions of outcome 4 between the CG (3.3%) and the HCDG (31.6%) and between the CG and the ADHDG (54.0%).

**Chart 2:** Description and comparison of APDQ outcomes according to study groups

**Variable**	**CG**		**HCDG**		**ADHDG**		
	**n**	**%**	**n**	**%**	**n**	**%**	**p-value** *
**Primary risk pooled**							<0.001
Outcome 1 (High risk of CAPD and Risk of CAPD)	1	3.3 a	10	26.3 ^a^	7	14.0	
Outcome 2 (High risk of ADHD and Risk of ADHD)	6	20.1	6	15.8	11	22.0	
Outcome 3 (Combined risk of CAPD/ADHD)	-	-	7	18.4	4	8.0	
Outcome 4 (Difficulty with language, learning, and listening)	1	3.3 b^,^c	12	31.6 ^b^	27	54.0 ^c^	
Outcome 5 (Language deficits)	1	3.3	2	5.3	1	2.0	
Outcome 6 (Normal listening)	21	70.0 d	1	2.6 ^d^	-	-	
**Total**	**30**	**100.0**	**38**	**100.0**	**50**	**100.0**	

* Fisher's Exact Test;

a,b,c,d Adjusted standardized residuals (values above 2.0 standard deviations indicate percentage differences)

**Caption:** APDQ = Auditory Processing Domains Questionnaire; CG = Control group; HCDG = Human communication disorders group; ADHDG = Attention Deficit Disorder group; ADHD = Attention Deficit Hyperactivity Disorder; CAPD = Central Auditory Processing Disorder; CI95% = 95% Confidence Interval

## DISCUSSION

The use of questionnaires in clinical practice has gained increasing prominence as these instruments can assist in screening individuals who may be at risk regarding auditory development^(2,6,7,13)^.

Regarding age range, in studies using the questionnaire, an increase in the total score in the "older children" group has been identified, a finding similar to that described in the literature^(17,19,23,37)^. In the present study, no statistically significant differences were observed between stratification by age range and the total APDQ score (Table 4). These results show that the total score on the questionnaire was not impacted by age, both in the total sample and in the analyzed groups, contradicting the hypothesis of the questionnaire author that younger children would have lower scores than older children due to the age difference. It is believed that this may be due to the reduced number of individuals in the "younger" (n = 64) and "older" (n = 54) groups in the present study when compared to the previous study (younger: n = 142; older: n = 126). Studies indicate that, as children get older, the maturation process of the central auditory nervous system occurs concomitantly with an increase in the level of attention, which may justify the improvement in scores with age progression^(38,39)^.

In the specialized literature, some studies also correlate responses to questionnaires investigating auditory processing abilities and found no correlation with the age effect^(39-42)^, corroborating the findings of the present research. In these mentioned studies, the age range of the participants varied more narrowly (between 6 and 12 years), unlike the present study, in which the age variation was greater. Even so, no developmental effect was observed. The sample size may have influenced this result, as the original study identified such an effect in a larger sample^(17)^.

In the questionnaire scoring, the CG participants presented median scores of 34, 41, and 100 points for the attention, language, and auditory processing domains, respectively (Table 5). This finding is similar to the national study conducted in 2022 with the previous 52-question questionnaire, in which the individuals were children and adolescents with normal hearing and presented median scores of 29, 43, and 114 points ^23.^ In general, the questionnaire presented similar results between the two Brazilian studies published to date.

A study conducted the application of the APDQ questionnaire in its Persian version to children with and without learning difficulties. The results presented by the authors were in percentages; however, high percentages were found in all domains studied for the CG when compared to the study group. The same occurs with the results of the questionnaire author, in which the percentages of the CG in three domains are higher than those of the other study groups, indicating that individuals with comorbidities present inadequate auditory abilities^(17,19)^.

In this study, individuals in the CG showed similar percentages to those in the original study^(17)^, with slightly higher values for the younger group in all three domains. Older individuals showed slightly lower percentages in the attention and auditory processing domains. In the HCDG, younger individuals performed better in the attention domain, while in the auditory processing domain, percentiles were lower than in the original study in both age groups^(17)^. In the language domain, although the values in the present study were slightly lower, they remained close to those of the original study^17.^ In the ADHDG, percentiles in the attention domain were quite similar to those of the original study^(17)^. Regarding auditory processing, younger individuals performed worse, while older individuals had similar results. In the language domain, both age groups showed lower percentiles compared to the original study^(17)^. When comparing these results, it is observed that the CG presented higher percentiles in relation to the original study, while the clinical groups showed lower percentiles, especially the ADHDG (Table 5).

Attention is a fundamental ability for various cognitive, linguistic, and auditory functions, including the proper execution of the peripheral and central auditory system assessments. Attention plays an essential role in the development of all auditory abilities, including auditory closure and figure-ground perception, allowing the individual to communicate clearly and efficiently. When inattention occurs, auditory abilities are compromised, also affecting communicative abilities, which generates more frequent complaints from caregivers^(43,44)^. Examples of area are questions 4 and 11, which are part of the auditory processing domain and are directly related to attention, which may help explain the reduced performance in the attention domains observed in both the HCDG and ADHDG. In the case of the ADHDG, this finding was expected as an initial hypothesis of the study.

In the HCDG, the domain with the worst performance was attention. This indicates that caregivers likely perceive, first and foremost, signs or symptoms related to inattention, without directly identifying difficulties in auditory processing. This suggests that impairment related to CAPD is not one of the parents' initial diagnostic hypotheses; they probably first associate attention and language difficulties with their children's impairment. It is also noteworthy that the questions in the attention domain address not only inattention, but also aspects such as hyperactivity and organization—as in questions 28 and 30—which may contribute to greater impairment in this domain. These results are unprecedented and present suggestive percentages for each of the domains among typically developing individuals, those with CAPD, and those with ADHD in the Brazilian population. To date, no other studies have used the 50-question version of the questionnaire for this comparative purpose.

Analyzing the APDQ questionnaire scores, the group with the best performance was the CG, while the ADHDG presented the worst scores (Table 5). When considering the educational level of the caregivers, it is noted that the CG caregivers have higher educational levels compared to those of the HCDG and ADHDG (Table 2). Based on these differences, it is hypothesized that the higher educational level of the caregivers in the CG may have positively influenced the scores of this group. This suggests that the understanding of the symptoms and the way the questionnaire was completed may be associated with the caregivers' level of education, potentially favoring a better recognition of the assessed signs. On the other hand, the lower educational level of the caregivers in the HCDG and ADHDG may have contributed to lower performance on the APDQ. Further studies should analyze this influence of the respondents' educational level on the APDQ to identify the signs and symptoms of behavioral alterations in their children.

A study indicated that parental education can exert a great influence on children's development, especially in cases of neurodevelopmental disorders, indicating that caregivers’ level of education contributes to the creation of a more stimulating environment, directly impacting the learning and development of children's cognitive and linguistic abilities, in addition to demonstrating the importance of considering the caregivers’ educational context when interpreting the results of the questionnaire^(45)^.

In the analysis between groups, a statistically significant difference was found between the CG and the HCDG, and between the CG and the ADHDG, both in the total questionnaire score and in the auditory processing, language, and attention domains. These findings indicate that the instrument is capable of differentiating children without complaints and/or alterations from those with complaints or difficulties across most questionnaire variables. However, the language and auditory processing domains did not differentiate individuals in the ADHDG from those in the HCDG, possibly due to the similarity of caregiver-reported complaints, and since both groups presented similar complaints related to oral and written language and listening difficulties. Given that these aspects are addressed in items included in both questionnaire domains and that CAPD diagnosis is intrinsically related to linguistic abilities, reduced scores in both domains were expected, thereby making differentiation between the two groups more challenging^(46,47)^.

However, the findings of the present study stand out for the attention domain, since the score in this domain could differentiate between ADHDG and HCDG (and vice versa). This finding has great scientific relevance and can be justified by the methodological rigor adopted in defining the inclusion and exclusion criteria of this research. The participants in the ADHDG were carefully selected, all with a multidisciplinary diagnosis of ADHD. In the HCDG, individuals with suspected comorbidities were excluded, based on screening using the SNAP-IV questionnaire, which also helped in differentiating between CAPD and ADHD. The questions regarding inattention and/or hyperactivity, important characteristics for ADHD diagnosis, are within the attention domain, confirming that the questions in the attention domain are efficient in encompassing the characteristics present in the ADHD diagnosis, in addition to contributing to studies that seek to identify differences between individuals with ADHD and other comorbidities, especially learning difficulties and CAPD^(48)^.

It is noteworthy that the use of the SNAP-IV as a criterion for identifying children and adolescents with signs and symptoms suggestive of ADHD has some limitations; however, it is widely used, both in research contexts and in clinical practice, due to its easy application and the support it offers to the diagnostic process^(49)^. The limitations of using the SNAP-IV are related to the fact that the questionnaire is completed by caregivers, which can lead to biases. Aspects such as education level and understanding of the described symptoms can influence the responses, affecting the questionnaire's outcome. Furthermore, the SNAP-IV may not correctly identify some symptoms, and a score that does not suggest an ADHD diagnosis does not necessarily eliminate the possibility of specialized assessment.

A study that managed to differentiate the individuals in the sample through the application of a questionnaire used LIFE – UK and observed a statistically significant difference between the performance of the CG and that of the other groups. In the research, the study group (children with complaints and a CAPD diagnosis) showed a greater number of symptoms related to hearing difficulties in the classroom when compared to the CG, presenting inferior performance and an indication of CAPD alteration^(40)^.

One of the distinguishing features of the APDQ questionnaire is its ability to identify and discriminate against the risks of individuals, assisting in referrals. In general, the questionnaire correctly identified 70% of individuals in the CG who did not present with complaints or a CAPD diagnosis and were classified in the normal listening outcome; that is, the questionnaire showed good specificity. For the HCDG, 97.4% of individuals presented outcomes indicating some risk of alteration. And in the ADHDG group, 100% of individuals presented a risk of alteration according to the APDQ screening (Chart 2).

In the analyses regarding the questionnaire outcomes, a statistically significant difference was found for outcomes 1, 4, and 6 between the CG and the HCDG (Chart 2). The questionnaire indicated that 70% of the CG presented normal listening (outcome 6), which corresponds to the expected result. For the HCDG, the highest percentage was for outcome 4, with 31.6%, followed by outcome 1, with 26.3%. This finding corroborates the difficulties encountered in the individuals, as they were individuals diagnosed with CAPD and learning difficulties. A similar result was also found for the ADHDG, which presented 54% for outcome 4, with a statistically significant difference, and 22% for outcome 2.

O'Hara and Mealings^(17)^, in their study with control groups, ADHD, CAPD, and learning disabilities, were able to correctly classify more than 80% of their subjects. A Persin study involving individuals with learning disabilities, identified 74.2% at risk of CAPD using the APDQ^(19)^. However, another group of researchers, also Persian, identified 3.3% of girls and 8.3% of boys at risk for CAPD. The authors report that the discrepancy in the results of the two studies may be due to the sample size and methodological criteria^(20)^. This result may be attributed to the fact that the authors used the previous version of the questionnaire with 50 questions, which only included three risk outcomes (high risk for CAPD, high risk for ADHD, and normal listening).

Diverse studies have identified a high percentage of participants at risk of CAPD. Research using the SAB questionnaire with children shows that, of the total sample, 36% of participants presented a CAPD risk, with 94.4% of these individuals showing alterations in one or more CAPD tests^(14)^. Another study with the same instrument involving 66 children found that 57.4% of the study population had scores below the established cutoff point, and all individuals presented three or more altered tests in the CAPD assessment. Both authors suggest that a low score on the SAB questionnaire indicates the possibility of CAPD alterations^(50)^. Research involving the QFISHER questionnaire to assess the auditory behavior of schoolchildren observed that 86.36% of the 22 participants were identified as having a risk of CAPD. The school performance category in the questionnaire showed the highest rate of alteration, reaching 87.72%. All studies suggest that using questionnaires can be an effective tool for identifying the risk of CAPD alterations, especially when associated with behavioral tests^(40)^.

Thus, it is noteworthy that the literature supports the idea that questionnaires and checklists are useful tools in identifying individuals who are at risk for auditory development. However, it is highlighted that screening tools are only one instrument for guiding conduct and cannot replace the clinical observation of a qualified professional and specialized assessment with clinical protocols based on scientific evidence^(2,6,7,13)^.

The data from this research are promising in differentiating individuals with hearing, attentional, and language difficulties. Based on the results, the use of the APDQ in a clinical context is suggested as a complementary tool in the CAP behavioral assessment in conjunction with anamnesis, and as a tool for comparing performance before and after auditory training. Furthermore, it may be useful in educational contexts, although its large-scale use may not be as practical due to the number of questions and the time required for application. However, its use can be valuable in differentiating specific students with similar and difficult-to-manage complaints, assisting in defining the most appropriate assessment to be performed. Thus, for individuals at risk for ADHD, referral for a speech-language pathology assessment and/or multidisciplinary assessment is suggested. For those at risk of language disorders, referral for a speech-language pathology assessment, with emphasis on oral and/or written language, is recommended. It is worth noting that, in these two cases, referrals should initially be made to the respective areas, and only after this process should the CAPD assessment be considered. Finally, for risk of CAPD, referral for CAPD assessment is suggested in order to investigate possible hearing difficulties.

This research had some limitations, such as the small number of participants in the CG compared to other international studies. This factor prevented more robust statistical analyses, which would have allowed for the definition of appropriate cut-off points for the investigated sample. Therefore, the cut-off points suggested by the questionnaire author were used. This can also be considered a limitation. Another limitation observed after data analysis concerns the educational level of the respondents, which varied among the groups formed in this study. Thus, the investigation recommends this care be taken in future studies. Another limitation of this study was the absence of a complete speech-language pathology assessment for the HCDG, which prevented a more detailed analysis. However, it should be noted that these individuals were carefully selected, with screening for possible comorbidities through questionnaires, as described in the methods.

In the specialized literature, the few studies using the APDQ discuss the questionnaire with 52 questions, making a direct comparison of the results of this research with those already published in scientific journals unfeasible.

It is worth highlighting that the results of this study reinforce the need for questionnaire-based research to incorporate factor analysis into its procedures. This technique plays a crucial role in structuring information, allowing the identification of key constructs or factors. By validating and refining research instruments, factor analysis increases the reliability and accuracy of measurements. Therefore, the application of factor analysis to the questionnaire under study is recommended as a fundamental means to improve the quality and relevance of the research findings. This should be one of the next steps to continue the APDQ studies at the national level.

Therefore, further studies are suggested, especially in Brazil, that explore the APDQ questionnaire (50 questions). Future research should also be conducted to compare its use with CAPD assessment and/or other screening questionnaires. Based on existing studies, the importance of using the APDQ in future research is emphasized due to its relevance and significant assistance in referrals for speech-language pathology diagnosis. This is also due to the lack of screening instruments that have undergone rigorous methodological analysis nationally. Furthermore, it is recommended that future studies include factor analyses and obtain adequate sensitivity and specificity values.

## CONCLUSION

In conclusion, the use of the APDQ demonstrated the ability to differentiate between different neurodevelopmental disorders in children and adolescents, both by total score and by questionnaire domains. A notable highlight was the attention domain, which proved effective in differentiating individuals with HCDG from those with ADHDG. The APDQ questionnaire outcomes helped identify individuals at risk of CAPD and/or ADHD. The results of this study are promising, highlighting the importance and need for further research to assess the effectiveness of the questionnaire in other study populations.

## Annex A. Auditory Processing Domains Questionnaire (APDQ) – Portuguese Version

**Table:**

**Auditory Processing Domains Questionnaire (APDQ)**				
**INFORMAÇÕES PESSOAIS**				
Nome da criança ___________________________________________________________________				
1.Data de hoje _________ 2.Data de nascimento da criança __________				
3. Sexo_________ 4. Ano escolar _______________				
5. Pessoa que responde ao questionário:				
(a) mãe _____________ (b) pai_______________ (c) outro membro da família (especifique) _____________ (d) professor _________ (e) outro (especifique) _____________________				
6. A língua utilizada na escola da criança é a língua materna da criança utilizada em casa? ( ) Sim ( ) Não				
7. Anos de escolaridade completos do pai ____________________________				
8. Anos de escolaridade completos da mãe ___________________________				
9. Assinale o grau de sua preocupação com as habilidades auditivas do estudante:				
(a) nenhum________ (b) leve__________ (c) moderada________ (d) alta_________				
10. Por favor, assinale a reação de sensibilidade / stress da criança a sons altos e ambientes ruidosos:				
(a) nenhuma________ (b) leve__________ (c) moderada________ (d) alta_________				
11. Por favor, assinale a dificuldade da criança em localizar sons (saber se um som está vindo da direita ou esquerda, de frente ou de trás, de perto ou de longe, de forma rápida ou lenta; se sabe quem está falando em um grupo ou de onde vem o latido de um cachorro):				
(a) nenhuma________ (b) leve__________ (c) moderada________ (d) alta_________				
12. Por favor, assinale quando uma ou mais das seguintes condições ou serviços ocorreram para esta criança:				
a.____ Educação Especial				
b.____ Dificuldade de Aprendizagem				
c.____ Transtorno Específico de Linguagem				
d.____ Dislexia (dificuldade de leitura)				
e.____ Histórico de atraso de aquisição de fala/linguagem ou terapia fonoaudiológica				
f._____ Perda auditiva permanente:				
(1) leve ________ (2) moderada ________ (3) severa __________				
(a) unilateral ______ (b) usa prótese auditiva ________ (c) implante coclear ________				
g.____ Aprendeu português como 2ª língua depois dos cinco anos				
h.____ Transtorno do Déficit de Atenção (TDAH)				
i._____ Otites médias crônicas ou de repetição ou Cirurgia (circule e explique) ________________________				
j._____ Icterícia ao nascimento:				
(a) leve________ (b) moderada_________ (c) severa ______ (d) transfusão sanguínea __________				
k.____ Transtorno do Processamento Auditivo (Central)				
l._____ Autismo / Síndrome de Asperger				
m.____ Atraso do desenvolvimento / retardo mental				
**Instruções:**				
Este questionário revisa as habilidades auditivas do dia-a-dia de um estudante. Linguagem, atenção e habilidades auditivas são importantes. Favor avaliar o desempenho do estudante em cada um dos itens abaixo baseado em suas observações. Lembre-se do que é esperado para ele ou ela na sua idade. O termo “ambientes ruidosos” refere-se aos ruídos de fundo de TV, vozes, música, máquinas, etc. Ruídos leves a moderados podem interferir na habilidade de ouvir palavras corretamente. “Ouvir corretamente” significa ouvir as declarações corretamente, sem precisar de repetições.				
Assinale:				
Coluna 1: se a habilidade for observada regularmente (mais de 75%)				
Coluna 2: se a habilidade for observada frequentemente (mais de 50%)				
Coluna 3: se a habilidade for observada algumas vezes (menos de 50%)				
Coluna 4: se a habilidade for observada raramente (menos de 25%)				
Avalie todos os itens – escreva N/A se for incapaz.				
	Quase sempre (mais de 75%)	Frequentemente (mais de 50%)	Às vezes (menos de 50%)	Raramente (menos de 25%)
1. Presta bem atenção quando conversa com uma única pessoa.				
2. Presta bem atenção ao ouvir em ambientes silenciosos na presença de outras pessoas (refeições, reuniões, aulas, etc).				
3. Presta bem atenção ao ouvir em ambientes ruidosos na presença de outras pessoas (refeições, reuniões, aulas, etc).				
4. Ouve suas palavras corretamente (sem repetições), quando presta atenção em ambientes silenciosos.				
5. Ouve suas palavras corretamente (sem repetições), quando presta atenção em ambientes ruidosos (onde outras pessoas podem estar falando ao mesmo tempo).				
6. Dedica um tempo para ouvir cuidadosamente e corretamente uma informação importante				
7. Compreende instruções quando presta atenção em ambientes silenciosos.				
8. Compreende instruções quando presta atenção em ambientes ruidosos.				
9. Compreende os outros quando está em locais em eco – academias, refeitórios, auditórios com auto falante.				
10. Entende a sua conversa enquanto outras pessoas falam ao lado (ex: em festas e refeições).				
11. Consegue te ouvir corretamente enquanto faz outra coisa (ex: vídeo - games ou pequenas tarefas domésticas).				
12. Consegue ouvir corretamente SEM pistas visuais (sem ver a face ou gestos do falante, sem ter figuras ou ilustrações).				
13. Concentra-se bem quando faz atividades que não exigem ouvir (estudar, outras tarefas domésticas).				
14. Concentra-se bem quando ouve histórias e apresentações.				
15. Compreende instruções escritas (conforme esperado para a idade).				
16. Cansa-se facilmente quando estuda (boceja ou brinca com as mãos).				
17. Cansa-se facilmente quando escuta (boceja ou brinca com as mãos).				
18. Consegue explicar coisas razoavelmente bem durante conversas.				
19. Concentra-se em tarefas importantes mesmo que não sejam divertidas ou interessantes.				
20. Ouve bem as palavras quando o falante está de costas (ou quando o falante está atrás da criança).				
21. Fala “o quê?” ou necessita de repetições quando conversa com interesse em ambientes silenciosos.				
22. Fala “o quê?” ou necessita de repetições quando conversa com interesse em ambientes ruidosos.				
23. Presta atenção a detalhes – evita erros por descuido quando faz a tarefa escolar.				
24. Compreende e usa frases mais longas (conforme esperado para a idade).				
25. Compreende e responde prontamente a perguntas em ambientes silenciosos (quando atento).				
26. Compreende e responde prontamente a perguntas em ambientes ruidosos (quando atento).				
27. Segue instruções orais, com etapas ou sequências (conforme esperado para a idade).				
28. Organiza tarefas e atividades para realiza-las em tempo.				
29. Compreende e usa gírias comuns para a idade.				
30. Perde ou esquece de fazer coisas (é avoado).				
31. Compreende pessoas que falam palavras de forma menos clara (rápido ou enrolado, com sotaques etc).				
32. Compreende falantes com vozes suaves ou agudas (pessoas tímidas – vozes infantis e algumas vozes femininas).				
33. Ouve bem o telefone sem precisar que a informação seja repetida (incluindo nomes e números).				
34. Consegue ouvir as pessoas corretamente a uma distância de 2 metros, aproximadamente (quando estão em pé ou sentadas juntas).				
35. Ouve errado ou confunde palavras com som parecido (como faca e vaca, sessenta e setenta).				
36. Lembra e usa novas palavras corretamente (conforme esperado para a idade).				
37. Consegue pronunciar palavras novas corretamente, após ouvi-las algumas vezes (incluindo nomes de pessoas e lugares).				
38. Consegue reconhecer os sons das letras e estabelecer a correspondência entre letra e a escrita de forma a auxiliar na leitura e na escrita correta da palavra (Conforme esperado para a idade).				
39. Lê em uma boa velocidade (conforme esperado para a idade).				
40. Controla impulsos e agitação a fim de evitar situações perigosas e que podem aborrecer.				
41. Lembra de detalhes de instruções ou pedidos feitos verbalmente (pouco tempo depois, sem a necessidade de repetição).				
42. Aprende bem as coisas ouvindo – sem precisar de mais explicações ou de apoio visual.				
43. Segue padrões rítmicos e de entonação batendo palmas, batucando ou cantarolando com os outros.				
44. Varia a própria voz para dar ênfase, falar com clareza e parecer mais agradável.				
45. Percebe “como” as coisas foram ditas ao interpretar comentários e seguir instruções (percebe diferentes tons de voz, ênfase em palavras chaves, etc).				
46. Compreende o que é falado sem precisar de palavras mais simples.				
47. Ouve bem sem precisar aumentar o volume das coisas (incluindo vozes e sinais de aviso).				
48. Consegue falar facilmente e fluentemente para a idade (sem esquecer palavras ou usar muitas pausas).				
49. Entende conversas e instruções sem precisar controlar os ruídos “extras” (ex: desligar a TV, fechar janelas, trocar de lugar).				
50. Entende as pessoas sem a necessidade de que falem mais devagar ou mais claramente.				
