# Supplementary material for: The use of the Auditory Processing Domains Questionnaire (APDQ) in Brazilian Portuguese with caregivers of children and adolescents with diverse neurodevelopmental conditions
Source: Codas. 2026 Mar 27;38(2):e20240347. doi: 10.1590/2317-1782/e20240347en (PMC13075843; doi:10.1590/2317-1782/e20240347en)
Supplement: Translation

# Translation: O uso do “Auditory Processing Domains Questionnaire” (APDQ) em português brasileiro em responsáveis de crianças e adolescentes com diferentes condições de neurodesenvolvimento

**DOI:** 10.1590/2317-1782/e20240347pt

**Authors:** Joel de Braga, Claudia Maria de Lorenzo, João Carlos Xikota, Luciany Andrade Nascimento, Liliane Desgualdo Pereira, Karin Ziliotto Dias, Maria Madalena Canina Pinheiro

**Affiliations:** 1 Programa de Pós-graduação em Fonoaudiologia, Universidade Federal de Santa Catarina – UFSC - Florianópolis (SC), Brasil.; 2 Núcleo Desenvolver , Hospital Universitário Polydoro Ernani de São Thiago, Empresa Brasileira de Serviços Hospitalares – EBSERH - Florianópolis (SC), Brasil.; 3 Departamento de Pediatria, Centro de Ciências da Saúde, Universidade Federal de Santa Catarina – UFSC - Florianópolis (SC), Brasil.; 4 Departamento de Fonoaudiologia, Universidade Federal de São Paulo – UNIFESP - São Paulo (SP), Brasil.; 5 Departamento de Fonoaudiologia, Universidade Federal de Santa Catarina – UFSC - Florianópolis (SC), Brasil.

**Keywords:** Percepção Auditiva, Inquéritos e Questionários, Audição, Transtorno do Déficit de Atenção com Hiperatividade, Criança, Adolescentes

## Abstract

**Objetivo:**

Investigar a capacidade do “Auditory processing domains questionnaire (APDQ)”, em sua versão português brasileiro, de diferenciar crianças e adolescentes com diferentes condições de neurodesenvolvimento com base nas respostas de seus responsáveis.

**Método:**

Estudo do tipo analítico e multicêntrico. Constituído por responsáveis de indivíduos entre sete e 17 anos de idade, distribuídos em três grupos em função da condição do neurodesenvolvimento, a saber: Grupo Controle (GC); Grupo Distúrbios da Comunicação Humana (GDCH) e Grupo Transtorno do Déficit de Atenção e Hiperatividade (GTDAH). Todos os responsáveis responderam o APDQ, que é uma escala composta por 50 questões reunidas em três domínios. O questionário gera um escore percentual para cada domínio, um total e a possibilidade de risco para alteração no desenvolvimento denominada como desfecho. Os dados foram analisados de forma descritiva e analítica, considerando um nível de significância de 5% (p<0,05).

**Resultados:**

Houve diferença significativa entre os grupos na pontuação total e nos domínios do APDQ, indicando que o instrumento consegue diferenciar crianças com e sem alterações do neurodesenvolvimento. No domínio de atenção, verificou-se diferença significativa entre GTDAH e GDCH, permitindo distinguir o desempenho desses grupos. Em relação aos desfechos, o APDQ conseguiu diferenciar os grupos entre si, classificando a maioria dos indivíduos do GC como “escuta normal” e os do GDCH e GTDAH como de risco para “dificuldade na linguagem, aprendizagem e escuta”.

**Conclusão:**

O uso do APDQ foi eficaz para distinguir as diferentes condições de neurodesenvolvimento. Destacou-se o domínio da atenção que permitiu diferenciar o GDCH e o GTDAH.

## INTRODUÇÃO

O Transtorno do Processamento Auditivo Central (TPAC) refere-se a alteração de uma ou mais habilidades auditivas, que pode acarretar prejuízos na compreensão da fala em ambientes ruidosos, na interpretação de ordens simples e complexas, bem como afetar aspectos não verbais, influenciando negativamente a aprendizagem, incluindo habilidades como leitura e escrita^(1,2)^.

Atualmente, a prevalência do TPAC em crianças é uma questão de considerável importância, com porcentagens variando em diferentes populações^(3,4)^. Nos Estados Unidos e no Reino Unido, a prevalência foi estimada entre 2-7%^(4,5)^, na Nova Zelândia, a prevalência geral na infância foi estimada em 6,2%^(6)^, enquanto estudos sugerem que 3-7% das crianças em idade escolar enfrentam dificuldades de aprendizagem^(3)^. Sendo assim, existe um número expressivo de encaminhamentos de crianças para avaliação^(6)^. Esse aumento nos encaminhamentos para avaliação pode ser atribuído a uma maior conscientização sobre o TPAC, ao uso de questionários subjetivos para rastreamento e às consequências que o transtorno pode acarretar na vida acadêmica e social das crianças.

Dessa forma, a identificação precoce do TPAC em crianças é fundamental para o rápido encaminhamento à avaliação e à intervenção, o que acelera o início do tratamento terapêutico, crucial para minimizar as dificuldades escolares e sociais^(2,6,7)^. Além disso, quanto mais cedo o TPAC é detectado, mais rapidamente podem ser implementadas modificações nos ambientes escolar e domiciliar, promovendo um contexto que favoreça o aprendizado, a compreensão e a comunicação da criança. Intervenções precoces também aproveitam a plasticidade cerebral, uma vez que o cérebro das crianças ainda está em processo de maturação, facilitando o desenvolvimento cognitivo e prevenindo o agravamento das dificuldades^(2,8,9)^.

Dentre os sinais e sintomas presentes no TPAC estão as dificuldades em prestar atenção ou evitar distrações, problemas de leitura, ortografia e/ou aprendizagem^(1)^ e entre as comorbidades, em especial, tem se destacado o Transtorno do Déficit de Atenção e Hiperatividade (TDAH). Estudos têm mostrado a importância da avaliação do processamento auditivo central (PAC) em crianças com TDAH, a fim de contribuir com o diagnóstico complementar e na reabilitação^(10,11)^. A relação entre o TDAH e o TPAC é significativa, visto que, geralmente, ambos os diagnósticos estão presentes de forma comórbida e se manifestam na primeira infância com queixas que envolvem atenção, memória, dificuldade em manter a concentração e em seguir instruções auditivas. Essa sobreposição de sintomas dificulta o diagnóstico de TPAC em crianças com TDAH, e vice-versa, na prática clínica. Portanto, pesquisas são realizadas com o intuito de estudar métodos de rastreio para a identificação precoce de TPAC em indivíduos de risco, além de auxiliar na diferenciação entre esses diagnósticos que podem apresentar sintomas semelhantes^(10,12)^.

No âmbito internacional, os *guidelines* apontam diversos instrumentos como forma de rastreio para detectar possíveis riscos e/ou manifestações comportamentais referente ao TPAC, tais como escalas, questionários e/ou bateria de testes auditivos. Essas ferramentas são potenciais na identificação de inabilidades auditivas nas mais diversas populações de estudo. Os documentos afirmam que os questionários fornecem informações sobre o comportamento auditivo e o seu impacto causado na comunicação, no desempenho acadêmico ou no trabalho, sendo fortemente sugeridos na prática clínica para identificação e intervenção precoce nas crianças com risco para o TPAC^(2,6,9,13)^.

Com relação à literatura brasileira, é possível encontrar, para o público infantil, questionários desenvolvidos informalmente sem validação; os traduzidos e validados; e os questionários somente traduzidos, sem dados referente à validação do instrumento em uma amostra significativa nas diferentes populações. Dentre esses instrumentos, pode-se citar o: “*Scale of Auditory Behaviors (SAB)* ^(14)^ *”*; “*Fisher’s auditory problems checklist (QFISHER)* ^(15)^ *”*; *Children's Auditory Processing Performance Scale* (CHAPS)^(16)^ e o “*Auditory Processing Domains Questionnaire (APDQ)* ^(17)^”.

Entre os questionários mencionados, o APDQ é relativamente recente na literatura e, até o momento, apresenta um número reduzido de publicações^(18-21)^. No entanto, o APDQ tem sido amplamente recomendado devido ao seu elevado rigor metodológico e ao adequado nível de sensibilidade e especificidade, tornando-o apropriado para aplicações em pesquisas^(22)^. Desenvolvido por O’Hara e Mealings^(17)^ para auxiliar no diagnóstico de TPAC, o APDQ permite identificar indivíduos com risco para TPAC ou com risco para outras comorbidades. O APDQ é um instrumento apropriado para que pais/responsáveis relatem sua percepção sobre a linguagem, a atenção e as habilidades auditivas cotidianas de indivíduos entre 7 e 17 anos^(17)^.

No Brasil, Dias et al.^(23)^ realizaram um estudo que envolveu a tradução, adaptação transcultural e avaliação da confiabilidade e validade interna do instrumento de 52 questões em indivíduos normo-ouvintes, sem fatores de risco para TPAC, na faixa etária de sete a 17 anos. Os resultados demonstraram que o instrumento apresenta qualidade de tradução, validade interna e confiabilidade favorável para seu uso^(23)^. Entretanto, em 2021, o autor do questionário, Brian O’Hara, revisou as perguntas e propôs a redução de duas questões do APDQ, resultando em um total de 50 questões^(24)^. A nova versão com 50 questões também foi analisada e publicada recentemente em um estudo brasileiro, que descreveu todas as etapas de tradução e adaptação do APDQ para o português brasileiro, apresentou as mudanças realizadas entre as duas versões do questionário — como a reformulação da escrita e a exclusão de algumas questões —, detalhou toda a metodologia para aplicação e manuseio da planilha e aplicou o instrumento em um pequeno grupo piloto^(25)^. Além deste, há estudo brasileiro que também utilizou a nova versão com indivíduos com Transtorno do Espectro Autista (TEA) Nível 1 e participantes neurotípicos^(26)^.

Considerando que o questionário APDQ diferencia aspectos de atenção e linguagem, acredita-se que a utilização da nova versão proposta pelo autor, que reduziu o número de questões de 52 para 50, trará uma grande contribuição clínica e científica. Isso auxiliará no processo de triagem, diagnóstico e reabilitação de crianças com TPAC, além de facilitar a identificação e diferenciação dos sinais e sintomas do TPAC em relação a outras comorbidades clínicas, como o TDAH. Assim, o objetivo do estudo foi investigar a capacidade do APDQ, em sua versão português brasileiro, de diferenciar crianças e adolescentes com diferentes condições de neurodesenvolvimento com base nas respostas de seus responsáveis.

## MÉTODO

Trata-se de um estudo do tipo observacional, analítico, transversal e multicêntrico, envolvendo três instituições do sul e sudeste do Brasil. Este estudo foi aprovado pelo Comitê de Ética em Pesquisa com Seres Humanos (CEPSH), sob número 5.268.520. Todos os responsáveis pelas crianças e/ou adolescentes foram orientados inicialmente sobre os objetivos do estudo e, após a explicação, assinaram o Termo de Consentimento Livre Esclarecido específico aos responsáveis legais dos menores, autorizando sua participação na pesquisa. Para os menores, foi solicitado que assinassem o Termo de Assentimento Livre e Esclarecido.

Este estudo dá continuidade às pesquisas sobre o APDQ em sua versão brasileira. Com a redução do instrumento para 50 questões e o aprimoramento da redação das perguntas, o questionário se tornou mais acessível e compreensível para o público-alvo. O artigo que detalha todo o processo de tradução, adaptação, aplicação e análise do APDQ do português brasileiro com suas 50 questões, está atualmente publicado em uma revista científica^(25)^.

### Definição da amostra

Trata-se de uma amostra por conveniência composta por três grupos. A seguir, descreve-se como cada grupo foi constituído e recrutado.

### Critérios de inclusão e exclusão

Os critérios de inclusão elencados para o estudo foram: responsáveis por indivíduos na faixa etária entre sete e 17 anos de idade, de ambos os sexos biológicos, com português-brasileiro como língua materna, ausência de patologias da orelha média, limiares auditivos dentro dos padrões de normalidade bilateralmente^(27)^.

Os critérios de exclusão foram: presença de relato dos responsáveis na anamnese de alterações neurológicas dos indivíduos da pesquisa e/ou observação dos avaliadores de alterações cognitivas que fossem evidentes.

Para o **Grupo Controle (GC)**, foram incluídos responsáveis por indivíduos sem histórico de alterações de orelha média na infância, bom desempenho escolar relatado em anamnese e ausência de diagnóstico de dislexia, TDAH ou qualquer outro transtorno do neurodesenvolvimento. Os participantes realizaram uma bateria mínima de testes comportamentais, na qual os resultados indicaram habilidades auditivas dentro do esperado para a faixa etária^(28,29)^. Além disso, os indivíduos do GC deveriam apresentar pontuação total no SAB maior ou igual a 46 pontos^(14)^ e, no SNAP-IV (*Swanson, Nolan, and Pelham Rating Scale*) não poderiam apresentar seis ou mais itens assinalados como 'bastante' e/ou 'demais' nos itens 1 a 9 e/ou 10 a 18^(30)^.

O **Grupo TDAH (GTDAH)** foi constituído por responsáveis de indivíduos com diagnóstico multidisciplinar de TDAH, podendo ser do tipo desatento, hiperativo ou combinado, confirmado por meio da avaliação multidisciplinar. Foram incluídos indivíduos que tinham pontuação no questionário SNAP-IV maior ou igual a 6 pontos nas questões de 1 a 9 ou de 10 a 18^(30)^. Além disso, poderiam apresentar em conjunto algum distúrbio da comunicação humana, relatado na anamnese com os responsáveis e pontuação no SAB menor que 46 pontos^(14)^ e/ou habilidades auditivas alteradas na avaliação do PAC.

No **Grupo Distúrbio da Comunicação Humana (GDCH)**, foram incluídos responsáveis de indivíduos que apresentavam queixas de leitura, escrita e/ou fala, e/ou alteração em ao menos um dos testes da bateria mínima do PAC^(28,29)^. Além disso, todos os indivíduos deveriam apresentar em conjunto algum distúrbio da comunicação humana (alterações de linguagem oral e/ou escrita), exceto o TDAH. Para eliminar sinais e sintomas sugestivos de TDAH, foram incluídos no GDCH apenas os responsáveis de indivíduos que não apresentavam pontuação no questionário SNAP-IV maior ou igual a 6 pontos nas questões de 1 a 9 ou nas questões de 10 a 18^(30)^. Além disso, deveriam apresentar pontuação no SAB menor que 46 pontos^(14)^.

### Avaliações

Todos os indivíduos da amostra realizaram uma bateria mínima de avaliação do PAC e foi considerada a presença de TPAC, quando havia alteração em uma ou mais habilidades auditivas avaliadas^(1,2,31)^. A bateria mínima foi elaborada com base nas recomendações e *guidelines* nacionais e internacionais, incluindo os seguintes testes^(1,2,31)^: **processamento temporal**: Random Gap Detection Test (RGDT)^(32)^ ou o Gaps in Noise (GIN)^(33)^ na avaliação da resolução temporal, sendo realizado o GIN em apenas apenas nove pacientes, sendo dois do GTDAH e sete do GDCH, pois eram adolescentes; e o teste padrão de frequência para avaliação da ordenação temporal, sendo a versão Auditec^(34)^ aplicada em indivíduos até 9 anos e 11 meses, e a versão Musiek^(35)^ para aqueles com 10 anos ou mais; **Reconhecimento de sons em tarefa dicótica**: teste dicótico de dígitos (integração e separação binaural)^(28)^; **Reconhecimento de sons verbais em tarefa monótica**: teste pediátrico de inteligibilidade de fala (PSI)^(28)^ ou pelo teste de identificação de sentenças sintéticas em escuta monótica (SSI)^(28)^, nas relações sinal/ruído de 0, -10 e -15 dB. O SSI foi aplicado aos indivíduos que apresentavam domínio de leitura fluente. Além da **avaliação simplificada** ^(28)^ para complementar a avaliação (localização sonora em cinco direções, teste de memória sequencial verbal e não verbal).

Além disso, os responsáveis de todos os indivíduos preencheram o questionário SNAP-IV^(30)^, que é um instrumento que oportuniza obter informações sobre a desatenção e/ou hiperatividade dos indivíduos, amplamente utilizado pelos profissionais e o questionário SAB^(14)^, um instrumento composto por 12 perguntas fechadas que investigam dificuldades auditivas comportamentais percebidas no cotidiano dos indivíduos.

### Divisão dos grupos

Com base nos critérios de inclusão e exclusão e nas avaliações aplicadas, os participantes foram distribuídos em três grupos:

**GC:** constituído por responsáveis de indivíduos com história de neurodesenvolvimento típico, com avaliação do PAC dentro dos padrões da normalidade para a faixa etária e ausência de queixas na anamnese e/ou nos questionários SAB^(14)^ e SNAP-IV^(30)^. Os membros do grupo foram recrutados pelos responsáveis pela pesquisa por meio de um formulário eletrônico divulgado em redes sociais e grupos de mensagens.
**GTDAH:** constituído por responsáveis de indivíduos com diagnóstico multidisciplinar de TDAH e com queixas relatadas em anamnese e nos questionários SAB^(14)^ e SNAP-IV^(30)^. Os participantes foram recrutados por meio de uma parceria estabelecida para fins de pesquisa com o ambulatório pediátrico de um hospital universitário no sul do país. Os indivíduos do ambulatório passaram por uma avaliação multidisciplinar e, após o diagnóstico, aqueles que preenchiam os critérios de inclusão eram convidados pelo pesquisador responsável a participar da pesquisa.
**GDCH:** constituído por responsáveis de indivíduos com queixas de alterações de fala, leitura e/ou escrita relatadas em anamnese e/ou no questionário SAB^(14)^ e habilidades auditivas alteradas. Os membros do grupo foram recrutados de duas instituições localizadas no sudeste do país, além de atendimentos realizados em um projeto de extensão e nas práticas de estágio de um curso de fonoaudiologia do sul do país.

### Aplicação do APDQ

O questionário possui três domínios que são analisados de forma complexa, permitindo identificar possíveis déficits de processamento auditivo, atenção e/ou linguagem no indivíduo avaliado, direcionando o encaminhamento para o profissional especializado (Anexo A).

O questionário foi aplicado pelos autores da pesquisa aos responsáveis pelos indivíduos e o tempo de aplicação do questionário foi de aproximadamente 30 minutos.

O APDQ aplicado neste estudo envolve 50 questões reunidas em três domínios: processamento auditivo com 29 itens; atenção com 10 itens e linguagem com 11 itens. Cada questão possui cinco opções de resposta com as seguintes pontuações: Quatro pontos para o comportamento que ocorre quase sempre; Três pontos para frequentemente; Um ponto se ocorre às vezes; Zero pontos se raramente o comportamento ocorre; “Não se aplica” não recebe nenhuma pontuação e a questão é excluída do cálculo final.

Os valores em porcentagem podem ser obtidos por meio do cálculo da seguinte fórmula:


$$Pontuação\;=\;\frac{pontos\;obtidos\;nas\;questões\;do\;determinado\;domínio}{4\;\times \;número\;de\;pontos\;máximo\;daquele\;domínio}\times 100$$ (1)

O máximo de pontos que pode ser obtido em cada domínio é de 116 pontos para processamento auditivo (29 questões), 40 pontos para atenção (10 questões) e 44 pontos para linguagem (11 questões), totalizando 200 pontos, o que sugere a ausência de um possível risco para os transtornos do neurodesenvolvimento. No entanto, a pontuação total do questionário pode chegar a 204 pontos, pois a questão 3 está presente em dois domínios, enquanto a questão 2 não está associada a nenhum dos domínios mas pode ser contabilizada na pontuação total.

O APDQ vem acompanhado de uma planilha do Microsoft Excel, denominada “*Database*”, disponível no site do autor do questionário, juntamente com um manual^(24)^. Esta planilha foi programada pelo autor do questionário para realizar os cálculos bastante complexos e gerar o relatório final, indicando a porcentagem em cada domínio e o risco primário de cada indivíduo, podendo ser classificados em um dos oito riscos: Alto risco de TPAC; Risco de TPAC; Alto risco de TDAH; Risco de TDAH; Risco combinado de TPAC e TDAH; Dificuldades na escuta, aprendizagem e linguagem; Déficits da linguagem e escuta normal.

### Interpretação do APDQ

No presente estudo, foram seguidos os pontos de corte (Quadro 1 e Tabela 1) estabelecidos no estudo original de Brian O'Hara e Mealings^(17)^. Os critérios 1) e 2) apresentados a seguir são os mesmos do estudo original do questionário, uma vez que, até o momento, não há estudos no Brasil que apresentem valores específicos para a população de crianças e adolescentes brasileiras na versão atual do questionário.

**Quadro 1:** Porcentagens e percentis de risco para alterações no Auditory Processing Domains Questionnaire

**Percentil**	**Faixa etária**	**Processamento auditivo**	**Atenção**	**Linguagem**
15° a 20°	7 a 10 anos	≤ 70%	≤ 60%	≤ 80%
Menor risco	11 a 17 anos	≤ 78%	≤ 67%	≤ 84%
5° a 10°	7 a 10 anos	≤ 56%	≤ 42%	≤ 72%
Maior risco	11 a 17 anos	≤ 62%	≤ 53%	≤ 78%

Fonte: O'Hara e Mealings^(17)^

**Tabela 1:** Síntese dos percentis necessários para a diferenciação dos riscos propostos pelo “Auditory Processing Domains Questionnaire”

**Risco primário**	**Percentil**			**Diferença entre ATT e PA**
	**PA**	**ATT**	**LGG**	
Alto risco de TPAC	≤ 5°	-	≥ 3°	≥ 0
Risco de TPAC	≤ 15	-	≥ 3°	≥ 0
Alto risco de TDAH	-	≤ 10* / ≤ 5**	≥ 3°	≤ -9
Risco de TDAH	-	≤ 20	≥ 3°	≤ -9
Risco combinado de TPAC e TDAH	≤ 15	≤ 20	≥ 3°	-1 a -8
Dificuldades na escuta, aprendizagem e linguagem	-	-	≤ 3°	-
Déficits da linguagem	-	-	3° a 15°	-
Escuta normal	> 15°	> 20	>15	-

* Percentil sugerido para indivíduos mais jovens (7 à 10 anos);

** Percentil sugerido para indivíduos mais velhos (11 a 17 anos)

**Legenda:** PA = Processamento auditivo; ATT = Atenção; LGG = Linguagem; TPAC = Transtorno do processamento auditivo central; TDAH = Transtorno do déficit de atenção e hiperatividade. Fonte: O'Hara e Mealings^(17)^

Para realizar a diferenciação dos possíveis riscos que o indivíduo pode apresentar, o questionário se baseia em dois critérios:

1. **Ponto de corte:** em cada domínio, existe um percentil que indica se o indivíduo apresenta risco e se esse risco é maior ou menor. Os valores estão organizados no Quadro 1.
2. **Diferença entre o domínio da atenção e o domínio do processamento auditivo:** essa diferença auxilia na identificação do risco que o indivíduo apresenta. A diferença entre as pontuações dos domínios de atenção e processamento auditivo sugere os seguintes riscos:

- Pontuações iguais ou acima de 0: Se a diferença entre as pontuações dos domínios de atenção e processamento auditivo for igual ou maior que 0, sugere que a pessoa pode estar enfrentando dificuldades relacionadas ao processamento auditivo.
- Pontuações entre -1 e -8: Se a diferença entre as pontuações dos domínios de atenção e processamento auditivo estiver entre -1 e -8, isso pode indicar fatores de risco combinados de TPAC e TDAH.
- Pontuações abaixo de -9: Se a diferença entre as pontuações dos domínios de atenção e processamento auditivo for menor do que -9, é um sinal de que o indivíduo pode estar em risco de TDAH.

O risco primário de linguagem é um caso especial, pois para que o indivíduo seja classificado com risco primário de déficit de linguagem, é necessário apresentar uma pontuação igual ou inferior a 45% (abaixo do 3º percentil), o que significa que estes indivíduos pontuaram muito baixo nas questões relacionadas à linguagem e assim o prejuízo neste domínio seria mais relevante. Isso bloqueia os riscos primários de TPAC e/ou TDAH, pois para que esses riscos sejam identificados, é preciso que o indivíduo obtenha uma pontuação suficiente no domínio de linguagem. Este é um aspecto importante ao se pensar nos encaminhamentos que serão priorizados a partir do resultado no APDQ.

Essas orientações presentes no manual do APDQ baseiam-se nos dados coletados pelo autor para a definição dos critérios de corte. A construção desses critérios envolveu a análise das respostas de 190 indivíduos do grupo controle, 20 estudantes com diagnóstico de transtorno do processamento auditivo, 45 estudantes com transtorno do déficit de atenção e 18 estudantes com transtornos de aprendizagem não especificados, todos frequentando aulas de educação especial^(17,24)^. Os resultados obtidos nos três domínios avaliados — processamento auditivo, linguagem e atenção — indicam os possíveis riscos primários identificados pelo APDQ.

Os pontos de corte foram definidos por meio de análises estatísticas, como a Curva Característica de Operação do Receptor (Curva ROC) e a regressão linear, que permitiram estabelecer percentuais e percentis associados a riscos de alteração. No estudo original, as análises de validade externa, realizadas com o modelo de regressão linear, revelaram diferenças estatisticamente significativas entre o grupo com desenvolvimento típico e os grupos clínicos em todas as escalas (p < 0,001), além de diferenças significativas entre os próprios grupos clínicos^(17)^. A Curva ROC identificou pontos de corte com níveis de sensibilidade e especificidade variando entre 80% e 90%. Os pontos de corte estabelecidos entre os percentis 15% e 20% priorizam a sensibilidade e são destacados em amarelo nos relatórios. Já os pontos de corte entre os percentis de 5% a 10% priorizam a especificidade e aparecem destacados em vermelho^(17)^.

A Tabela 1 resume os percentis necessários para que o questionário diferencie os indivíduos em cada um dos oito possíveis riscos primários do APDQ.

### Análise estatística

Para as variáveis categóricas da amostra, os dados foram representados por meio de frequências absolutas e relativas. As variáveis numéricas foram descritas através de medidas de dispersão.

Em relação à faixa etária, os indivíduos foram estratificados em dois grupos, conforme a proposta do autor do questionário: crianças de 7 a 10 anos foram classificadas como “crianças mais jovens”, e aquelas de 11 a 17 anos como “crianças mais velhas”.

As variáveis quantitativas foram testadas quanto à normalidade usando o teste Shapiro-Wilk, assimetria, curtose e histogramas. Quando a distribuição era normal, aplicou-se o teste t para amostras independentes. Caso contrário, usaram-se os testes não paramétricos de Mann-Whitney e Kruskal-Wallis. Quando o teste de Kruskal-Wallis indicou diferença estatisticamente significativa (p < 0,05), foi realizada uma análise post-hoc de Dunn para comparações múltiplas. Consideraram-se significativas apenas as comparações que também apresentaram significância no post-hoc^(36)^.

Na análise dos oito desfechos, dois deles foram agrupados para evitar uma dispersão excessiva dos dados, onde cada item apresentaria valores muito pequenos. Assim, “Alto risco de TPAC” e “risco de TPAC” foram combinados no desfecho 1; “Alto risco de TDAH” e “Risco de TDAH” formaram o desfecho 2; “Risco combinado de TPAC e TDAH” formou o desfecho 3; “Dificuldade na linguagem, aprendizagem e escuta” formou o desfecho 4; “Déficits de linguagem” formou o desfecho 5 e “Escuta normal” o desfecho 6. Para avaliar a diferença dos seis desfechos entre os grupos, utilizou-se o teste Exato de Fisher. Quando encontrada associação, a diferença entre as categorias foi investigada pelos resíduos padronizados ajustados, cujo valor acima de 2,0 desvios-padrão indicou as cédulas/caselas com diferença entre os percentuais e sinalizados na tabela com as letras “a, b, c, d”. Os dados foram armazenados em planilhas do programa Microsoft Excel e posteriormente exportados para o *software* Stata, versão 14.0. Para as análises estatísticas, foi considerado um nível de significância de 5% (p < 0,05).

## RESULTADOS

Na composição dos indivíduos de acordo com os grupos, foi observado que 19 indivíduos foram excluídos por não atenderem aos critérios de inclusão. Ao final, 118 indivíduos foram selecionados, sendo que a maioria (54,2%) tinha entre 7 e 10 anos (crianças mais jovens), 58,5% eram do sexo masculino e 17,79% estavam cursando o 5º ano do ensino fundamental.

A Tabela 2 apresenta os dados que caracterizam a amostra em relação aos dados sociodemográficos e inabilidades auditivas de acordo com os grupos do estudo. Cabe ressaltar que não foi possível obter informações sobre a escolaridade de quatro responsáveis, pois o familiar que estava respondendo não tinha conhecimento a respeito desta informação. Em relação à avaliação do PAC, verificou-se que todos os indivíduos do GTDAH apresentaram pelo menos uma das habilidades auditivas alteradas, sendo que esse grupo apresentou um número maior de inabilidades auditivas em comparação ao GDCH.

**Tabela 2:** Caracterização da amostra de acordo com os dados sócio-demográficos e inabilidades auditivas

**Variáveis**	**GC (n = 30)**	**GDCH (n = 38)**	**GTDAH (n = 50)**
**Idade (anos)**			
Med	9,5	11	10
Min- Max (DP)	7 - 16 (2,7)	7 - 17 (2,7)	7 - 17 (2,1)
**Sexo**			
M (n - %)	13 (44%)	22 (57%)	34 (68%)
F (n - %)	17 (56%)	16 (43%)	16 (32%)
N° de inabilidades auditivas			
1 a 2 inabilidades	0	8 (21,06%)	6 (12%)
3 a 4 inabilidades	0	14(36,84%)	22(44%)
5 ou mais inabilidades	0	16 (42,10)%)	22(44%)
**Escolaridade do pai**			
Ensino fundamental (n - %)	1 (3,3%)	12 (32,43%)	15 (31,91%)
Ensino médio (n - %)	10 (33,3%)	14 (37,85%)	30 (63,85%)
Ensino superior (n - %)	14 (46,7%)	9 (24,32%)	1 (2,12%)
Pós - graduação (n - %)	5 (16,7%)	2 (5,40%)	1 (2,12%)
**Escolaridade da mãe**			
Ensino fundamental (n - %)	0	4 (10,54%)	12 (24%)
Ensino médio (n - %)	7 (24,1%)	21 (55,26%)	25 (50%)
Ensino superior (n - %)	17 (58,7%)	10 (26,31%)	10 (20%)
Pós - graduação (n - %)	5 (17,2%)	3 (7,89%)	3 (6%)

**Legenda:** GC = Grupo Controle; GDCH = Grupo Distúrbios da Comunicação Humana; GTDAH = Grupo Transtorno do Déficit de Atenção e Hiperatividade; N = número de participantes; Med = Média; M = Masculino; F = Feminino; % = porcentagem; Mínimo; Max = máximo; DP = desvio padrão

Na Tabela 3, apresentamos o desempenho dos três grupos na bateria mínima do PAC, apenas para caracterizar a amostra e complementar os dados. Observou-se que, na maioria dos testes, o GTDAH teve o pior desempenho, seguido pelo GDCH e pelo GC.

**Tabela 3:** Análise descritiva da bateria mínima do processamento auditivo central segundo os grupos de estudo e controle

**Teste comportamentais**			**GC**		**GTDAH**		**GDCH**	
			**Média (DP)**	**Mediana**	**Média (DP)**	**Mediana**	**Média (DP)**	**Mediana**
**Localização sonora (n)**			4,73 (0,44)	5	4,48 (0,73)	**5**	4,71 (0,56)	**5**
**Teste de memória sequencial verbal (n)**			2,96 (0,18)	3	1,90 (1,02)	2	2,23 (0,75)	2
**Teste de memória sequencial não verbal (n)**			2,73 (0,44)	3	2,09 (0,85)	2	2,28 (0,83)	2,5
**RGDT (ms)**			6,04 (2,97)	5,75	64,50 (95,63)	22,50	65,13 (101,14)	14
**GIN (ms)**	**OD**		**-**	**-**	7,5 (6,36)	7,5	6,85 (3,18)	5
	**OE**		**-**	**-**	10 (7,07)	10	6,85 (2,73)	6
**Teste dicótico de dígitos - integração (%)**	**OD**		97,17 (0 04)	100	89,11 (11,02)	93,13	91,25 (8,66)	94,38
	**OE**		96,92 (0,04)	97,50	86,33 (11,93)	87,50	90 (7,70)	93
**Teste dicótico de dígitos - separação (%)**	**OD**		94,08 (0,07)	95	82,98 (12,38)	87,50	83,41 (15,43)	85
	**OE**		92,83 (0,07)	95	75,59 (16,09)	16,09	77,16 (19,43)	82,50
**Teste Padrão de Frequência - Auditec (%)**	**Nomeação**		91,55 (0,07)	93,33	62 (32,35)	73	74 (20,29)	77
	**Humming**		**-**	**-**	54 (36,06)	65	79 (13,26)	80
**Teste Padrão de Frequência - Musiek (%)**	**Nomeação**		91,68 (0,08)	93,33	44 (33,53)	46	53,84 (30,35)	60
	**Humming**		-	-	53,84 (30,35)	60	51,10 (27,65)	77
**Teste pediátrico de inteligibilidade de fala - PSI (%)**	**OD**	S/R (0)	93,48 (0,77)	100	89 (12,46)	90	90 (15,21)	100
		S/R (-10)	89,09 (0,10)	90	83 (15,01)	80	84,24 (15,01)	90
		S/R (-15)	75,83 (0,10)	75	65 (17,96)	60	66,54 (23,82)	70
**Teste pediátrico de inteligibilidade de fala - PSI (%)**	**OE**	S/R (0)	93,21 (0,07)	100	90 (9,62)	90	87,58 (17,14)	90
		S/R (-10)	92,73 (0,08)	90	87 (14,89)	90	86,67 (16,14)	90
		S/R (-15)	73,33 (0,12)	75	76 (19,83)	70	81,54 (25,41)	95
**Teste de identificação de sentenças sintéticas - SSI (%)**	**OD**	S/R (0)	91,67 (0.09)	95	80 (12,25)	80	72,50 (22,17)	70
		S/R (-10)	90 (0,10)	90	82 (17,22)	85	66 (28,81)	60
		S/R (-15)	78,33 (0,07)	80	70 (18,71)	70	53,33 (32,15)	40
	**OE**	S/R (0)	91,67 (0,11)	95	76 (18,17)	70	70 (25,82)	70
		S/R (-10)	91,67 (0,11)	95	82 (19,41)	85	58 (27,75)	60
		S/R (-15)	86,67 (0,10)	90	86 (13,42)	86	73,33 (20,82)	80

**Legenda:** n = número absoluto; % = porcentagem; ms = milissegundos; OD = Orelha direita; OE = Orelha esquerda; DP = Desvio padrão; GC = Grupo controle; GTDAH = Grupo Transtorno do Déficit de Atenção e Hiperatividade; GDCH = Grupo Distúrbio da Comunicação Humana; RGDT = random gap detection test; Gaps in noise (GIN); R/S = relação sinal/ruído

Na Tabela 4, são apresentadas as pontuações totais do APDQ, segundo a divisão de faixas etárias dos indivíduos cujos respondentes compuseram os grupos de estudo. Os indivíduos foram estratificados em “crianças mais jovens” e “crianças mais velhas”, no entanto, não foram observadas diferenças estatisticamente significantes entre a divisão por faixa etária e a pontuação do questionário, tanto para a amostra total quanto para os grupos de estudo.

**Tabela 4:** Descrição da pontuação total do APDQ entre as faixas etárias dos indivíduos de acordo com os grupos do estudo e amostra total

**Variável**	**Pontuação total do APDQ**		
	**Crianças mais jovens (7 a 10)**	**Crianças mais velhas (11 a 17)**	**p-valor**
**GC *(n=30)***	n = 20	n = 10	0,643^a^
Média (DP)	174,4 (26,8)	166,1 (36,1)	
Mediana (IIQ)	180,5 (162 – 191,5)	181 (164 – 182)	
**GDCH *(n=38)***	n = 17	n = 21	0,511^b^
Média (DP)	106,1 (46,8)	114,9 (34,1)	
Mediana (IIQ)	124 (72 – 132)	117 (80 – 144)	
**GTDAH *(n=50)***	n = 26	n = 24	0,144^b^
Média (DP)	83,6 (24,5)	95,4 (31,6)	
Mediana (IIQ)	77 (64 – 97)	91 (70 – 123)	
**Total da amostra *(n=118)***	n = 63	n = 55	0,963^a^
Média (DP)	118,0 (50,7)	116,0 (41,6)	
Mediana (IIQ)	114 (74,5 – 167,5)	116,5 (80 – 147)	

a Teste Mann-Whitney;

b Test t de Student

**Legenda:** DP = Desvio padrão; IIQ = Intervalo Interquartil; APDQ = *Auditory Processing Domains Questionnaire*; GC = Grupo Controle; GDCH = Grupo Distúrbio da Comunicação Humana; GTDAH = Grupo Transtorno de Déficit de Atenção e Hiperatividade; N = número de participantes

Na Tabela 5 e na Figura 1 será apresentada a pontuação total do APDQ segundo os domínios em cada um dos grupos. Observa-se que o grupo controle apresentou medianas mais elevadas, tanto na pontuação total do APDQ quanto nos diversos domínios do instrumento, quando comparado aos GDCH e GTDAH, sendo esta disparidade estatisticamente significante (p = 0,001) no teste de Kruskal-Wallis.

**Tabela 5:** Análise da pontuação total e do domínios do APDQ por grupo

**Domínios**	**GC (n=30)**	**GDCH (n=38)**	**GTDAH (n=50)**	**p-valor**	**p-valor** *
**Pontuação total**				**< 0,001**	
Med (DP)	171 (29,9)	111 (40,0)	89 (28,3)		**a,b - < 0,001** c > 0,079
Mediana (IIQ)	181 (164 - 188)a,b	119 ( 76 - 143)a,b,c	84,5 (66 - 108)a,b,c		
**Atenção**				**< 0,001**	
Med (DP)	31,8 (7,28)	22,3 (8,3)	15,4 (7,6)		**a,b - < 0,001**
Mediana (IIQ)	34 (29 - 36)a,b	24 (16 -28)a,b,c	14 (11 - 21)a,b,c		**c > 0,005**
Percentil					
7 a 10 anos (mais jovens)	84%	47%	24%		
11 a 17 anos (mais velhos)	78%	53%	28%		
**Linguagem**				**< 0,001**	
Med (DP)	39,2 (6,9)	24,2 (10,2)	20,6 (8,2)		**a,b - < 0,001**
Mediana (IIQ)	41 (38 - 44)a,b	25,5 (18 - 30)a,b,c	18,5 (15 -25)a,b,c		c > 0,306
Percentil					
7 a 10 anos (mais jovens)	99%	56%	42%		
11 a 17 anos (mais velhos)	92%	55%	46%		
**Processamento auditivo**				**< 0,001**	
Med (DP)	96,8 (17,3)	62,2 (23,5)	51,4 (17,5)		**a,b - < 0,001**
Mediana (IIQ)	100 (92 - 108)a,b	66 (40 - 77)a,b,c	48,5 (38 - 64)a,b,c		c > 0,154
Percentil					
7 a 10 anos (mais jovens)	87%	55%	40%		
11 a 17 anos (mais velhos)	86%	54%	47%		

Teste de Kruskal-Wallis

* Teste *post-hoc* de Dun

**Legenda:** APDQ = *Auditory Processing Domains Questionnaire;* n = número de participantes; GC = Grupo Controle; GDCH = Grupo Distúrbios da comunicação humana; GTDAH = Grupo Transtorno do Déficit de Atenção e Hiperatividade; Med = média; DP = desvio padrão; IIQ= Intervalo Interquartil

**Figura 1:** Descrição da pontuação total e dos domínios do APDQ

**Legenda:** Med = mediana; A = grupo controle; B = grupo distúrbio de comunicação humana; C = grupo TDAH. *Teste de Kruskal-Wallis

Durante a realização do teste *post hoc* para comparações entre grupos de acordo com a pontuação total e domínios, constatou-se que as diferenças foram estatisticamente significantes nas comparações entre o GTDAH e o GC (p = 0,001), bem como entre o GDCH e o GC (p = 0,001). Não foram identificadas diferenças significativas entre o GTDAH e GDCH na pontuação total do questionário e nem nos domínios da linguagem e de processamento auditivo (p > 0,05). A única exceção foi observada no domínio da atenção, no qual houve diferença entre os dois grupos (p = 0,005), indicando que, nesse domínio, o questionário conseguiu não apenas distinguir o grupo controle dos grupos de estudo, mas também diferenciou os dois grupos de estudo entre si.

Quanto aos valores percentuais do APDQ apresentados na Tabela 5, observa-se que o GC apresentou, tanto entre os mais jovens quanto entre os mais velhos, os maiores valores nos três domínios. O domínio da linguagem apresentou as maiores porcentagens, seguido pelo processamento auditivo e, por último, a atenção, com os menores valores. No GDCH, os domínios de processamento auditivo e linguagem mostraram porcentagens semelhantes e ligeiramente superiores às do domínio da atenção. Já no GTDAH, os domínios de processamento auditivo e linguagem apresentaram valores próximos entre si, porém com uma diferença mais acentuada em relação ao domínio da atenção, que apresentou os menores valores. Em relação às porcentagens, o GC não apresentou risco de alteração, ao contrário dos demais grupos, que, quando comparados aos valores de corte (Quadro 1), apresentaram escores em percentil de risco para alteração.

No Quadro 2 estão descritos os desfechos do APDQ de acordo com os grupos. Observa-se que, no grupo controle, 70% dos casos exibiram um desfecho 6. Para o GDCH, a maior proporção da amostra foi classificada com desfecho 4 (31,6%), seguido do desfecho 1 (26,3%). No GTDAH, as maiores ocorrências foram o desfecho 4 (54,0%) e o desfecho 2 (22%). Houve diferença nas proporções do desfecho 1 (3,3% *vs* 26,3%) e desfecho 6 (70,0% *vs* 2,6%) entre o GC e o GDCH. Também foi observada diferença nas proporções do desfecho 4 entre o GC (3,3%) e o GDCH (31,6%) e entre o GC e o GTDAH (54,0%).

**Quadro 2:** Descrição e comparação dos desfechos do APDQ de acordo com os grupos do estudo

**Variável**	**GC**		**GDCH**		**GTDAH**		
	**n**	**%**	**n**	**%**	**n**	**%**	**p-valor** *
**Risco primário agrupado**							<0,001
Desfecho 1 (Alto risco de TPAC e Risco de TPAC)	1	3,3^a^	10	26,3a	7	14,0	
Desfecho 2 (Alto risco de TDAH e Risco de TDAH)	6	20,1	6	15,8	11	22,0	
Desfecho 3 (Risco combinado de TPAC/TDAH)	-	-	7	18,4	4	8,0	
Desfecho 4 (Dificuldade na linguagem, aprendizagem e escuta)	1	3,3^b,^c	12	31,6b	27	54,0^c^	
Desfecho 5 (Déficits de linguagem)	1	3,3	2	5,3	1	2,0	
Desfecho 6 (Escuta normal)	21	70,0d	1	2,6^d^	-	-	
**Total**	**30**	**100,0**	**38**	**100,0**	**50**	**100,0**	

* Teste Exato de Fisher;

a,b,c,d Resíduos padronizados ajustados (valores acima de 2,0 desvios - padrão indicou diferença percentuais)

**Legenda:** APDQ = *Auditory Processing Domains Questionnaire*; GC = Grupo controle; GDCH = Grupo distúrbios da comunicação humana; GTDAH = Grupo Transtorno de Déficit de Atenção; TDAH = Transtorno de Déficit de Atenção; TPAC = Transtorno do Processamento Auditivo Central; IC95% = Intervalo de Confiança de 95%

## DISCUSSÃO

A utilização de questionários na prática clínica tem ganhado cada vez mais destaque, pela capacidade que os instrumentos possuem de auxiliar no rastreio de indivíduos que possam apresentar riscos referente ao desenvolvimento auditivo^(2,6,7,13)^.

Em relação à faixa etária, nas pesquisas com o questionário, é possível identificar aumento da pontuação total do questionário na faixa etária “crianças mais velhas”, achado semelhante ao descrito na literatura^(17,19,23,37)^. No presente estudo, não foram observadas diferenças estatisticamente significantes entre a estratificação por faixa etária e a pontuação total do APDQ (Tabela 4). Esses resultados mostram que a pontuação total no questionário não foi influenciada pela idade, tanto na amostra total quanto nos grupos analisados, contrariando a hipótese do autor do questionário de que as crianças mais jovens teriam resultados inferiores aos das crianças mais velhas devido à diferença de idade. Acredita-se que isso possa ser devido ao número reduzido de indivíduos no grupo “mais jovens” (n = 64) e “mais velhos” (n = 54) no presente estudo quando comparado ao estudo anterior (mais jovens: n = 142; mais velhos: n = 126). Estudos referem que, com o avanço da idade, ocorre o processo de maturação do sistema nervoso auditivo central concomitantemente ao aumento do nível da atenção, podendo justificar a melhora da pontuação com a progressão de idade^(38,39)^.

Na literatura especializada, encontram-se trabalhos que também correlacionam as respostas de questionários que investigam as habilidades auditivas e não encontraram correlação com o efeito idade^(39-42)^, corroborando com os achados da presente pesquisa. Nos estudos mencionados, a faixa etária dos participantes variou de forma mais restrita (entre 6 e 12 anos), diferentemente do presente estudo, em que a variação etária foi maior. Ainda assim, não se observou efeito do desenvolvimento. Acredita-se que o tamanho da amostra possa ter influenciado esse resultado, uma vez que o estudo original identificou tal efeito em uma população maior^(17)^.

Na pontuação do questionário, os participantes do GC apresentaram a mediana de 34, 41 e 100 pontos para os domínios de atenção, linguagem e processamento auditivo, respectivamente (Tabela 5). Tal achado assemelha-se ao estudo nacional realizado em 2022 com o questionário anterior de 52 questões, no qual os indivíduos eram crianças e adolescentes normo-ouvintes e apresentaram medianas de 29, 43 e 114 pontos^(23)^. De forma geral, o questionário apresentou resultados semelhantes entre as duas pesquisas brasileiras publicadas até o momento.

Um estudo realizou a aplicação do questionário APDQ em sua versão persa em crianças com e sem dificuldades de aprendizagem. Os resultados trazidos pelos autores foram em porcentagens; porém, é possível observar porcentagens elevadas em todos os domínios estudados para o GC quando comparado ao grupo estudo. O mesmo ocorre com os resultados do autor do questionário, em que as porcentagens dos três domínios do GC são maiores do que as dos demais grupos de estudo, indicando que indivíduos com comorbidades apresentam habilidades auditivas inadequadas^(17,19)^.

No presente estudo, os indivíduos do GC apresentaram percentuais semelhantes aos do estudo original^(17)^, com valores ligeiramente maiores para o grupo mais jovem nos três domínios. Já os mais velhos, observaram-se percentuais ligeiramente menores nos domínios de atenção e processamento auditivo. No GDCH, os indivíduos mais jovens tiveram desempenho superior no domínio da atenção, enquanto no domínio do processamento auditivo, os percentis foram inferiores aos do estudo original em ambas as faixas etárias^(17)^. No domínio da linguagem, embora os valores do presente estudo tenham sido ligeiramente menores, ainda se mantiveram próximos ao do estudo original^(17)^. No grupo GTDAH, os percentis no domínio da atenção foram bastante semelhantes aos do estudo original^(17)^. Em relação ao processamento auditivo, os indivíduos mais jovens apresentaram desempenho inferior, ao passo que os mais velhos tiveram resultados aproximados. Já no domínio da linguagem, ambas as faixas etárias apresentaram percentis mais baixos em comparação ao estudo original^(17)^. Ao comparar esses resultados, observa-se que o GC apresentou percentis mais altos em relação ao estudo original, enquanto os grupos clínicos mostraram percentis inferiores, especialmente o grupo com TDAH (Tabela 5).

A atenção é uma habilidade fundamental para diversas funções cognitivas, linguísticas e auditivas, inclusive para a execução adequada das avaliações do sistema auditivo periférico e central. Ela desempenha um papel essencial no desenvolvimento de todas as habilidades auditivas, inclusive para o fechamento auditivo e figura-fundo, o que permite que o indivíduo se comunique com clareza e eficiência. Quando há desatenção, ocorre um comprometimento nas habilidades auditivas, afetando também as habilidades comunicativas, o que gera queixas mais frequentes por parte dos responsáveis^(43,44)^. Exemplos disso são as questões 4 e 11, que fazem parte do domínio do processamento auditivo e estão diretamente relacionadas à atenção, o que pode ajudar a explicar o desempenho reduzido no domínios da atenção, observado tanto no GDCH quanto no GTDAH. No caso do GTDAH, esse achado já era esperado como hipótese inicial do estudo.

No GDCH, o domínio com pior desempenho foi a atenção. Isso indica que os responsáveis possivelmente percebem, em primeiro lugar, sinais ou sintomas relacionados à desatenção, sem identificar diretamente dificuldades no processamento auditivo. Isso sugere que o prejuízo relacionado ao TPAC não é uma das hipóteses de diagnóstico inicial dos pais, que provavelmente associam primeiro as dificuldades de atenção e linguagem ao prejuízo de seus filhos. Vale destacar ainda que as questões do domínio da atenção abordam não apenas desatenção, mas também aspectos como hiperatividade e organização — como é o caso das questões 28 e 30 — o que pode contribuir para o maior comprometimento neste domínio. Esses resultados são inéditos e apresentam percentuais sugestivos para cada um dos domínios entre indivíduos típicos, com TPAC e com TDAH na população brasileira. Até o momento, não há outros estudos que tenham utilizado a versão de 50 questões do questionário com esse objetivo comparativo.

Analisando as pontuações do questionário APDQ, observa-se que o grupo com melhor desempenho foi o GC, enquanto o GTDAH apresentou as piores pontuações (Tabela 5). Quando se considera também a escolaridade dos responsáveis, nota-se que os responsáveis pelo GC têm níveis de escolaridade mais elevados em comparação aos dos grupos GDCH e GTDAH (Tabela 2). Com base nessas diferenças, é possível hipotetizar que a maior escolaridade dos responsáveis pelo grupo GC pode ter influenciado positivamente as pontuações desse grupo. Isso sugere que a compreensão dos sintomas e a forma de preenchimento do questionário podem estar associadas ao nível de instrução dos responsáveis, potencialmente favorecendo um melhor reconhecimento dos sinais avaliados. Por outro lado, a menor escolaridade dos responsáveis pelos grupos GDCH e GTDAH pode ter contribuído para um menor desempenho no APDQ. Novos estudos devem analisar essa influência do nível de escolaridade dos respondentes no APDQ para identificar os sinais e sintomas de alterações comportamentais de seus filhos(as).

Um estudo indicou que a escolaridade dos pais pode exercer uma grande influência no desenvolvimento das crianças, especialmente em casos de alterações no neurodesenvolvimento, indicando que a formação dos responsáveis contribui para a criação de um ambiente mais estimulante, impactando diretamente o aprendizado e o desenvolvimento das habilidades cognitivas e linguísticas das crianças, além de demonstrar a importância de considerar o contexto educacional dos responsáveis ao interpretar os resultados do questionário^(45)^.

Na análise entre os grupos, verificou-se diferença estatisticamente significativa entre o GC e o GDCH, e entre o GC e o GTDAH, tanto na pontuação total do questionário quanto nos domínios de processamento auditivo, linguagem e atenção. Esses achados indicam que o instrumento é capaz de diferenciar o grupo de crianças sem queixas e/ou alterações das crianças com queixas/dificuldades em grande parte das variáveis do questionário. Já os domínios da linguagem e processamento auditivo não conseguiram diferenciar os indivíduos do GTDAH e GDCH, possivelmente devido à similaridade das queixas relatadas pelos responsáveis e visto que os dois grupos possuem queixas semelhantes relacionadas à linguagem oral, escrita e dificuldades de escuta. Sabendo que estes temas são inseridos em questões presentes nos dois domínios do questionário e que o diagnóstico de TPAC tem uma relação intrínseca com as habilidades linguísticas, era esperado que a pontuação nos dois domínios fosse reduzida, tornando a diferenciação entre os dois grupos mais difícil^(46,47)^.

Entretanto, destaca-se os achados do presente estudo para o domínio da atenção, uma vez que a pontuação neste domínio conseguiu diferenciar o GTDAH e o GDCH (vice e versa). Esse achado tem grande relevância científica e pode ser justificado pelo rigor metodológico adotado na definição dos critérios de inclusão e exclusão desta pesquisa. Os participantes do GTDAH foram cuidadosamente selecionados, todos com diagnóstico multidisciplinar de TDAH. Já no GDCH, foram excluídos os indivíduos com suspeita de comorbidades, mediante o rastreio realizado pelo questionário SNAP-IV, que também auxiliou na diferenciação entre TPAC e TDAH. As questões referentes a desatenção e/ou hiperatividade, características importantes para o diagnóstico de TDAH, estão dentro do domínio da atenção, confirmando que as perguntas presentes no domínio da atenção são eficientes para abranger as características presentes no diagnóstico de TDAH, além de contribuir para estudos que buscam identificar diferenças entre indivíduos com TDAH e as demais comorbidades, em especial, as dificuldades de aprendizagem e TPAC^(48)^.

É importante destacar que o uso do SNAP-IV como critério para identificar crianças e adolescentes com sinais e sintomas sugestivos de TDAH possui algumas limitações, no entanto, é amplamente utilizado, tanto em contextos de pesquisa quanto na prática clínica, devido à sua facilidade de aplicação e ao suporte que oferece ao processo diagnóstico^(49)^. As limitações do uso do SNAP-IV estão relacionadas ao fato de que o preenchimento do questionário é realizado pelos responsáveis, o que pode levar a vieses. Aspectos como o nível de escolaridade e a compreensão sobre os sintomas descritos podem influenciar as respostas fornecidas, afetando o resultado do questionário. Além disso, é possível que o SNAP-IV não identifique corretamente alguns sintomas, e uma pontuação que não sugere o diagnóstico de TDAH não necessariamente elimina a possibilidade de avaliação especializada.

Na literatura, é possível encontrar um estudo que conseguiu diferenciar os indivíduos da amostra por meio da aplicação de um questionário, o estudo em questão utilizou o LIFE – UK e observou diferença estatisticamente significativa entre o desempenho do GC e o dos demais grupos. Na pesquisa, o grupo estudo (crianças com queixas e diagnóstico de TPAC) sinalizou um maior número de sintomas referentes às dificuldades auditivas em sala de aula quando comparado ao GC, apresentando desempenho inferior e indicação de alteração de PAC^(40)^.

Um dos diferenciais do questionário APDQ é sua habilidade em identificar e discriminar os riscos dos indivíduos, auxiliando nos encaminhamentos. De forma geral, o questionário conseguiu identificar corretamente 70% dos indivíduos do GC, que não apresentavam queixa nem diagnóstico de TPAC e foram classificados no desfecho de escuta normal; ou seja, o questionário apresentou uma boa especificidade. Já para o GDCH, 97,4% dos indivíduos apresentaram desfechos que indicam algum risco de alteração. E no GTDAH, 100% dos indivíduos apresentaram risco de alteração de acordo com o rastreio do APDQ (Quadro 2).

Nas análises referentes aos desfechos do questionário, houve diferença estatística para os desfechos 1, 4 e 6 entre o GC e GDCH (Quadro 2). O questionário apontou que 70% do GC apresentava escuta normal (desfecho 6), o que corresponde ao esperado. Para o GDCH, a porcentagem maior foi para o desfecho 4, com 31,6%, seguido do desfecho 1, com 26,3%. Tal achado corrobora as dificuldades encontradas nos indivíduos, visto que eram indivíduos com diagnóstico de TPAC e dificuldades na aprendizagem. Resultado similar também foi encontrado para o GTDAH, que apresentou 54% para o desfecho 4, com diferença estatisticamente significante e 22% para o desfecho 2.

O’Hara e Mealings (2018), em seu estudo com os grupos controle, TDAH, TPAC e de dificuldade de aprendizagem, conseguiram classificar corretamente mais de 80% dos seus indivíduos^(17)^.Os autores do estudo persa com indivíduos que apresentavam dificuldade de aprendizagem, identificaram por meio do APDQ, 74,2% para risco de TPAC^(19)^. Entretanto, outro grupo de pesquisadores, também persa, identificou 3,3% de meninas e 8,3% de meninos com risco para o TPAC. Os autores relatam que a discrepância do resultado dos dois estudos pode ser devido ao número da amostra e aos critérios metodológicos^(20)^. Acredita-se que esse resultado possa ser atribuído ao fato de os autores terem utilizado a versão anterior do questionário com 50 questões, a qual contemplava somente três desfechos de risco (alto risco para TPAC, alto risco para TDAH e escuta normal).

Na literatura, é possível encontrar diferentes estudos que identificaram um alto percentual de participantes em risco de alterações do PAC. Pesquisas com o questionário SAB e crianças demonstram que, da amostra total, 36% dos participantes apresentaram risco de alteração no PAC, sendo 94,4% desses indivíduos apresentando alterações em um ou mais testes do PAC^(14)^. Outro estudo com o mesmo instrumento envolvendo 66 crianças constatou que 57,4% da população de estudo apresentava pontuação abaixo do ponto de corte estabelecido e todos esses indivíduos apresentaram três ou mais testes alterados na avaliação do PAC. Ambos os autores sugerem que uma pontuação baixa no questionário SAB indica a possibilidade de alterações no PAC^(50)^. Uma pesquisa envolvendo o questionário QFISHER para avaliar o comportamento auditivo de escolares observou que 86,36% dos 22 participantes foram identificados como tendo risco de alteração no PAC. A categoria de desempenho escolar no questionário apresentou a maior taxa de alteração, atingindo 87,72%. Todos os estudos sugerem que a utilização de questionário pode ser uma ferramenta eficaz para identificar o risco de alterações no PAC, especialmente quando associados a testes comportamentais^(40)^.

Sendo assim, é notório observar que a literatura possui dados que concretizam a ideia de que os questionários e *checklist* são ferramentas úteis na identificação dos indivíduos que possuem riscos para o desenvolvimento auditivo. Porém, é importante ressaltar que as ferramentas de triagens são apenas um instrumento de direcionamento de conduta, não podendo excluir o olhar clínico do profissional capacitado e da avaliação especializada com protocolos clínicos embasados em evidências científicas^(2,6,7,13)^.

Os dados desta pesquisa são promissores na diferenciação de indivíduos com dificuldades auditivas, atencionais e de linguagem. Com base nos resultados obtidos, sugere-se o uso do APDQ em contexto clínico como uma ferramenta complementar na avaliação comportamental do PAC, em conjunto com a anamnese e como uma ferramenta de comparação de desempenho pré e pós treinamento auditivo. Além disso, pode ser útil em contextos educacionais, embora seu uso em larga escala possa não ser tão prático devido ao número de questões e ao tempo necessário para a aplicação. No entanto, sua utilização pode ser valiosa para diferenciar escolares específicos, com queixas semelhantes e de difícil manejo, auxiliando na definição da avaliação mais adequada a ser realizada. Dessa forma, para indivíduos com risco para TDAH, sugere-se encaminhamento para uma avaliação fonoaudiológica e/ou avaliação multidisciplinar. Para aqueles com risco de alterações na linguagem, recomenda-se encaminhamento para avaliação fonoaudiológica, com ênfase na linguagem oral e/ou escrita. Vale ressaltar que, nesses dois casos, os encaminhamentos devem ser feitos inicialmente para as respectivas áreas, e somente após esse processo, considerar a avaliação do PAC. Por fim, para risco de TPAC, sugere-se encaminhamento para avaliação do PAC a fim de investigar possíveis dificuldades auditivas.

Na presente pesquisa existiram algumas limitações, tais como: o número reduzido de participantes no GC quando comparado aos demais estudos internacionais. Esse fator impediu a realização de análises estatísticas mais robustas, que possibilitariam a definição de pontos de corte próprios para a amostra investigada. Diante disso, optou-se por utilizar os pontos de corte sugeridos pelo autor do questionário. Isso pode ser considerado também uma limitação. Outra limitação verificada após a análise dos dados diz respeito ao nível de escolaridade dos respondentes que variou entre os grupos formados neste estudo. Assim recomenda-se que em futuros estudos esse cuidado seja tomado. Outra limitação deste estudo foi a ausência de avaliação fonoaudiológica completa para os participantes do GDCH, o que impossibilitou uma análise mais detalhada. Ressalta-se, contudo, que esses indivíduos foram cuidadosamente selecionados, com rastreamento de possíveis comorbidades por meio de questionários, conforme descrito nos métodos.

Na literatura especializada os poucos estudos com o APDQ discutem o questionário com 52 questões, tornando inviável a comparação direta dos resultados da presente pesquisa com os já publicados nas revistas científicas.

Vale ressaltar que, os resultados deste estudo reforçam a necessidade de que pesquisas baseadas em questionários passem a incorporar a análise fatorial em seus procedimentos. Essa técnica desempenha um papel crucial na estruturação das informações, permitindo a identificação dos principais construtos ou fatores. Ao realizar a validação e o refinamento dos instrumentos de pesquisa, a análise fatorial aumenta a confiabilidade e precisão das medições. Portanto, recomenda-se a aplicação da análise fatorial ao questionário em estudo, como um meio fundamental para aprimorar a qualidade e relevância das descobertas da pesquisa. Esta deverá ser uma das próximas etapas que dão continuidade aos estudos do APDQ no âmbito nacional.

Assim sugere-se a realização de mais estudos, principalmente brasileiros que explorem o questionário APDQ (50 questões). E também pesquisas futuras que relacionem o seu uso comparativamente com a avaliação de PAC e/ou outros questionários de rastreio. Com base nos estudos existentes enfatiza-se a importância da utilização do APDQ em pesquisas futuras, devido a sua relevância e significativo auxílio nos encaminhamentos para o diagnóstico fonoaudiológico. E, também devido à carência de instrumentos de rastreio que tenham sido submetidos a uma análise metodológica rigorosa no âmbito nacional. E ainda, recomenda-se incluir nos futuros estudos a presença de análises fatoriais e a obtenção de valores de sensibilidade e especificidade adequados.

## CONCLUSÃO

Conclui-se que o uso do APDQ demonstrou capacidade de diferenciar as diferentes condições de transtorno de neurodesenvolvimento de crianças e adolescentes tanto pela pontuação total quanto pelos domínios do questionário. Um destaque notável foi o domínio da atenção, que se mostrou eficaz na diferenciação dos indivíduos do GDCH em relação ao GTDAH. Os desfechos do questionário APDQ auxiliaram na identificação de indivíduos em risco de TPAC e/ou TDAH. Os resultados deste estudo são promissores, ressaltando a importância e a necessidade de pesquisas adicionais para avaliar a eficácia do questionário em outras populações de estudo.

## Anexo A. “Auditory Processing Domains Questionnaire (APDQ)”

**Table:**

**Auditory Processing Domains Questionnaire (APDQ)**				
**INFORMAÇÕES PESSOAIS**				
Nome da criança ___________________________________________________________________				
1.Data de hoje _________ 2.Data de nascimento da criança __________				
3. Sexo_________ 4. Ano escolar _______________				
5. Pessoa que responde ao questionário:				
(a) mãe _____________ (b) pai_______________ (c) outro membro da família (especifique) _____________ (d) professor _________ (e) outro (especifique) _____________________				
6. A língua utilizada na escola da criança é a língua materna da criança utilizada em casa? ( ) Sim ( ) Não				
7. Anos de escolaridade completos do pai ____________________________				
8. Anos de escolaridade completos da mãe ___________________________				
9. Assinale o grau de sua preocupação com as habilidades auditivas do estudante:				
(a) nenhum________ (b) leve__________ (c) moderada________ (d) alta_________				
10. Por favor, assinale a reação de sensibilidade / stress da criança a sons altos e ambientes ruidosos:				
(a) nenhuma________ (b) leve__________ (c) moderada________ (d) alta_________				
11. Por favor, assinale a dificuldade da criança em localizar sons (saber se um som está vindo da direita ou esquerda, de frente ou de trás, de perto ou de longe, de forma rápida ou lenta; se sabe quem está falando em um grupo ou de onde vem o latido de um cachorro):				
(a) nenhuma________ (b) leve__________ (c) moderada________ (d) alta_________				
12. Por favor, assinale quando uma ou mais das seguintes condições ou serviços ocorreram para esta criança:				
a.____ Educação Especial				
b.____ Dificuldade de Aprendizagem				
c.____ Transtorno Específico de Linguagem				
d.____ Dislexia (dificuldade de leitura)				
e.____ Histórico de atraso de aquisição de fala/linguagem ou terapia fonoaudiológica				
f._____ Perda auditiva permanente:				
(1) leve ________ (2) moderada ________ (3) severa __________				
(a) unilateral ______ (b) usa prótese auditiva ________ (c) implante coclear ________				
g.____ Aprendeu português como 2ª língua depois dos cinco anos				
h.____ Transtorno do Déficit de Atenção (TDAH)				
i._____ Otites médias crônicas ou de repetição ou Cirurgia (circule e explique) ________________________				
j._____ Icterícia ao nascimento:				
(a) leve________ (b) moderada_________ (c) severa ______ (d) transfusão sanguínea __________				
k.____ Transtorno do Processamento Auditivo (Central)				
l._____ Autismo / Síndrome de Asperger				
m.____ Atraso do desenvolvimento / retardo mental				
**Instruções:**				
Este questionário revisa as habilidades auditivas do dia-a-dia de um estudante. Linguagem, atenção e habilidades auditivas são importantes. Favor avaliar o desempenho do estudante em cada um dos itens abaixo baseado em suas observações. Lembre-se do que é esperado para ele ou ela na sua idade. O termo “ambientes ruidosos” refere-se aos ruídos de fundo de TV, vozes, música, máquinas, etc. Ruídos leves a moderados podem interferir na habilidade de ouvir palavras corretamente. “Ouvir corretamente” significa ouvir as declarações corretamente, sem precisar de repetições.				
Assinale:				
Coluna 1: se a habilidade for observada regularmente (mais de 75%)				
Coluna 2: se a habilidade for observada frequentemente (mais de 50%)				
Coluna 3: se a habilidade for observada algumas vezes (menos de 50%)				
Coluna 4: se a habilidade for observada raramente (menos de 25%)				
Avalie todos os itens – escreva N/A se for incapaz.				
	Quase sempre (mais de 75%)	Frequentemente (mais de 50%)	Às vezes (menos de 50%)	Raramente (menos de 25%)
1. Presta bem atenção quando conversa com uma única pessoa.				
2. Presta bem atenção ao ouvir em ambientes silenciosos na presença de outras pessoas (refeições, reuniões, aulas, etc).				
3. Presta bem atenção ao ouvir em ambientes ruidosos na presença de outras pessoas (refeições, reuniões, aulas, etc).				
4. Ouve suas palavras corretamente (sem repetições), quando presta atenção em ambientes silenciosos.				
5. Ouve suas palavras corretamente (sem repetições), quando presta atenção em ambientes ruidosos (onde outras pessoas podem estar falando ao mesmo tempo).				
6. Dedica um tempo para ouvir cuidadosamente e corretamente uma informação importante				
7. Compreende instruções quando presta atenção em ambientes silenciosos.				
8. Compreende instruções quando presta atenção em ambientes ruidosos.				
9. Compreende os outros quando está em locais em eco – academias, refeitórios, auditórios com auto falante.				
10. Entende a sua conversa enquanto outras pessoas falam ao lado (ex: em festas e refeições).				
11. Consegue te ouvir corretamente enquanto faz outra coisa (ex: vídeo - games ou pequenas tarefas domésticas).				
12. Consegue ouvir corretamente SEM pistas visuais (sem ver a face ou gestos do falante, sem ter figuras ou ilustrações).				
13. Concentra-se bem quando faz atividades que não exigem ouvir (estudar, outras tarefas domésticas).				
14. Concentra-se bem quando ouve histórias e apresentações.				
15. Compreende instruções escritas (conforme esperado para a idade).				
16. Cansa-se facilmente quando estuda (boceja ou brinca com as mãos).				
17. Cansa-se facilmente quando escuta (boceja ou brinca com as mãos).				
18. Consegue explicar coisas razoavelmente bem durante conversas.				
19. Concentra-se em tarefas importantes mesmo que não sejam divertidas ou interessantes.				
20. Ouve bem as palavras quando o falante está de costas (ou quando o falante está atrás da criança).				
21. Fala “o quê?” ou necessita de repetições quando conversa com interesse em ambientes silenciosos.				
22. Fala “o quê?” ou necessita de repetições quando conversa com interesse em ambientes ruidosos.				
23. Presta atenção a detalhes – evita erros por descuido quando faz a tarefa escolar.				
24. Compreende e usa frases mais longas (conforme esperado para a idade).				
25. Compreende e responde prontamente a perguntas em ambientes silenciosos (quando atento).				
26. Compreende e responde prontamente a perguntas em ambientes ruidosos (quando atento).				
27. Segue instruções orais, com etapas ou sequências (conforme esperado para a idade).				
28. Organiza tarefas e atividades para realiza-las em tempo.				
29. Compreende e usa gírias comuns para a idade.				
30. Perde ou esquece de fazer coisas (é avoado).				
31. Compreende pessoas que falam palavras de forma menos clara (rápido ou enrolado, com sotaques etc).				
32. Compreende falantes com vozes suaves ou agudas (pessoas tímidas – vozes infantis e algumas vozes femininas).				
33. Ouve bem o telefone sem precisar que a informação seja repetida (incluindo nomes e números).				
34. Consegue ouvir as pessoas corretamente a uma distância de 2 metros, aproximadamente (quando estão em pé ou sentadas juntas).				
35. Ouve errado ou confunde palavras com som parecido (como faca e vaca, sessenta e setenta).				
36. Lembra e usa novas palavras corretamente (conforme esperado para a idade).				
37. Consegue pronunciar palavras novas corretamente, após ouvi-las algumas vezes (incluindo nomes de pessoas e lugares).				
38. Consegue reconhecer os sons das letras e estabelecer a correspondência entre letra e a escrita de forma a auxiliar na leitura e na escrita correta da palavra (Conforme esperado para a idade).				
39. Lê em uma boa velocidade (conforme esperado para a idade).				
40. Controla impulsos e agitação a fim de evitar situações perigosas e que podem aborrecer.				
41. Lembra de detalhes de instruções ou pedidos feitos verbalmente (pouco tempo depois, sem a necessidade de repetição).				
42. Aprende bem as coisas ouvindo – sem precisar de mais explicações ou de apoio visual.				
43. Segue padrões rítmicos e de entonação batendo palmas, batucando ou cantarolando com os outros.				
44. Varia a própria voz para dar ênfase, falar com clareza e parecer mais agradável.				
45. Percebe “como” as coisas foram ditas ao interpretar comentários e seguir instruções (percebe diferentes tons de voz, ênfase em palavras chaves, etc).				
46. Compreende o que é falado sem precisar de palavras mais simples.				
47. Ouve bem sem precisar aumentar o volume das coisas (incluindo vozes e sinais de aviso).				
48. Consegue falar facilmente e fluentemente para a idade (sem esquecer palavras ou usar muitas pausas).				
49. Entende conversas e instruções sem precisar controlar os ruídos “extras” (ex: desligar a TV, fechar janelas, trocar de lugar).				
50. Entende as pessoas sem a necessidade de que falem mais devagar ou mais claramente.
